# Selenium sulfide disrupts the PLAGL2/C‐MET/STAT3‐induced resistance against mitochondrial apoptosis in hepatocellular carcinoma

**DOI:** 10.1002/ctm2.536

**Published:** 2021-09-15

**Authors:** Tianfeng Yang, Jian Huo, Rui Xu, Qi Su, Wenjuan Tang, Dongdong Zhang, Man Zhu, Yingzhuan Zhan, Bingling Dai, Yanmin Zhang

**Affiliations:** ^1^ School of Pharmacy Health Science Center Xi'an Jiaotong University Xi'an P. R. China; ^2^ State Key Laboratory of Shaanxi for Natural Medicines Research and Engineering Xi'an P. R. China

**Keywords:** C‐MET/STAT3, HCC, PLAGL2, selenium sulfide

## Abstract

**Background:**

Hepatocellular carcinoma (HCC) is the third leading cause of cancer‐related deaths worldwide. Overexpression of pleomorphic adenoma gene like‐2 (PLAGL2) is associated with tumorigenesis. However, its function in HCC is unclear, and there are currently no anti‐HCC drugs that target PLAGL2. Drug repositioning may facilitate the development of PLAGL2‐targeted drug candidates.

**Methods:**

The expression of PLAGL2 in HCC clinical tissue samples and HCC cell lines was analyzed by western blotting. The constructed HCC cell models were used to confirm the underlying function of PLAGL2 as a therapeutic target. Multiple in vitro and in vivo assays were conducted to determine the anti‐proliferative and apoptosis‐inducing effects of selenium sulfide (SeS_2_), which is clinically used for the treatment of seborrheic dermatitis and tinea versicolor.

**Results:**

PLAGL2 expression was higher in HCC tumor tissues than in normal adjacent tissues. Its overexpression promoted the resistance of HCC cells of mitochondrial apoptosis through the regulation of the downstream C‐MET/STAT3 signaling axis. SeS_2_ exerted significant anti‐proliferative and apoptosis‐inducing effects on HCC cells in a PLAGL2‐dependent manner. Mechanistically, SeS_2_ suppressed C‐MET/STAT3, AKT/mTOR, and MAPK signaling and triggered Bcl‐2/Cyto C/Caspase‐mediated intrinsic mitochondrial apoptosis both in vitro and in vivo.

**Conclusions:**

Our data reveal an important role of PLAGL2 in apoptosis resistance in HCC and highlight the potential of using SeS_2_ as a PLAGL2 inhibitor in patients with HCC.

## Graphical abstract

The C‐MET/STAT3 signaling axis as a novel downstream target of pleomorphic adenoma gene like‐2 (PLAGL2) contributed to PLAGL2‐induced mitochondrial apoptosis resistance in HCC. Selenium sulfide (SeS_2_) induced cell proliferation inhibition and apoptosis on HCC cells by inhibiting the activity of PLAGL2 and restraining the novel PLAGL2/C‐MET/STAT3, AKT/mTOR and MAPK signaling pathways.

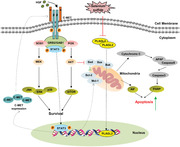

**Abbreviations**
ERK: extracellular regulated protein kinase
IHC: immunohistochemistry
MAPK: mitogen‐activated protein kinase
mTOR: mechanistic target of rapamycin
STAT3: signal transducer and activator of transcription 3

## 1 INTRODUCTION

Hepatocellular carcinoma (HCC), the most common form of liver cancer, is the third leading cause of cancer‐related mortality worldwide, and its incidence presents a gradually increasing trend.^1^ Surgical, locoregional and systemic therapies are available for HCC, but they are hampered by rigorous clinical selection criteria and a high frequency of disease recurrence.^2^ Although molecular targeted drug therapies are widely used and have demonstrated significant effects, the prognosis of patients with HCC remains poor; thus, it is important to develop new anti‐HCC treatments.^3^ The mechanisms underlying HCC progression are largely unknown; therefore, it is essential to identify optional therapeutic targets and develop novel effective drugs to improve the treatment outcomes in HCC.

Pleomorphic adenoma gene like‐2 (PLAGL2), a member of the PLAG family of proteins, is a zinc‐finger transcription factor.^4^ The *PLAGL2* gene encodes a protein comprising 496 amino acids. PLAGL2 contains six zinc fingers and is usually located in the nucleus.^5^ It has been reported that PLAGL2 contributes to tumorigenesis and the development of a wide variety of different tumors. For instance, PLAGL2 overexpression is associated with lung cancer progression, where advanced stages of lung cancer are associated with a higher PLAGL2 expression.^6^ In addition, PLAGL2 is positively correlated with the degree of tumor invasion in gastrointestinal cancer and colorectal cancer.^7^, ^8^ In HCC, PLAGL2 can regulate the EMT‐related Wnt/β‐catenin and EGFR/AKT signaling pathways.^9^ Furthermore, PLAGL2 and Pirh2 dimers can negatively regulate the levels and stability of p53.^10^ In neuroblastoma, PLAGL2 induces cell cycle regulation and apoptosis by activating the Nip3 promoter independent of HIF‐1;^11^ however, the mechanism underlying PLAGL2‐mediated apoptosis regulation in HCC is not yet fully understood. In addition, no clinically approved drugs targeting PLAGL2 are available to date. Therefore, it would be valuable to study the role of PLAGL2 in HCC and explore potential drugs that can target it.

The expression of mesenchymal‐epithelial transition factor (C‐MET) is commonly upregulated in various cancers.^12^ The abnormal activation of C‐MET plays crucial roles in cancer cell proliferation and the resistance of programmed apoptosis.^13^ The binding of intracellular adapter proteins to C‐MET leads to the activation of specific cascades, such as STAT3, AKT/mTOR, and MAPK signaling cascades.^14^ However, numerous C‐MET inhibitors, including the well‐known drugs cabozantinib and capmatinib, have failed in clinical trials involving HCC patients.^15^ These data highlight the significance of clarifying the role of the intracellular regulator of C‐MET in HCC.

Drug repositioning offers a relatively shorter approval period and a simpler path to clinical translation than traditional drug structural design and high‐throughput screening.^16^ Selenium sulfide (SeS_2_) is a clinical agent used for the treatment of seborrheic dermatitis and tinea versicolor.^17^, ^18^ Several studies have shown that Se exerts anti‐cancer effects in addition to supplying substrates for selenoprotein synthesis, such as sodium selenite.^18^, ^19^ However, the potential mechanism of action of SeS_2_ in HCC growth suppression has not been reported to date. Therefore, based on the principle of drug repositioning, we aimed to discover new functions of SeS_2_ and further investigated the anti‐HCC effect and the underlying mechanism of action of SeS_2_. Here, we found that PLAGL2 expression was upregulated in HCC tumor tissues and that PLAGL2 overexpression substantially promoted apoptosis resistance in HCC cells. Moreover, we found that the C‐MET/STAT3 signaling axis acted as a novel downstream target of PLAGL2 and contributed to the inhibition of PLAGL2‐mediated proliferation and apoptosis induction of SeS_2_ in HCC in vitro and in vivo. Our findings suggest that PLAGL2 plays a vital role in HCC apoptosis resistance and supports the use of SeS_2_ as a promising PLAGL2 inhibitor for HCC therapy.

## 2 MATERIALS AND METHODS

### 2.1 Chemicals and reagents

The details of the reagents used in this study are listed in Table S1. For western blotting analysis, all primary antibodies except GAPDH were used at a dilution of 1:1000, and GAPDH and goat anti‐rabbit IgG secondary antibodies were used at a dilution of 1:10000.

### 2.2 Cell lines and cell culture

The human normal hepatocyte cell lines L‐02 and the HCC cell lines Hep3B and Huh‐7 were purchased from the Shanghai Institute of Cell Biology at the Chinese Academy of Sciences (Shanghai, China). The HCC cell lines SMMC‐7721, Bel‐7402, Bel‐7404, SK‐Hep‐1, and HepG2 were obtained from Genechem Co., Ltd. (Shanghai, China). The HCC cell line MHCC‐97L was obtained as a gift from the First Affiliated Hospital of Xi'an Jiaotong University. MHCC‐97L, Huh‐7, and HepG2 cells were cultured in DMEM containing 10% FBS. L‐02, SMMC‐7721, Bel‐7402, and Bel‐7404 cells were cultured in RPMI‐1640 medium, while Hep3B and SK‐Hep‐1 cells were cultured in MEM medium containing 10% FBS. All media were supplemented with penicillin (100 U/ml) and streptomycin (100 U/ml). All cells were maintained in an incubator with a humidified atmosphere of 5% CO_2_ at 37°C.

### 2.3 Patient tissues

HCC patient tissues were acquired from the First Affiliated Hospital of Xi'an Jiaotong University. To avoid multi‐factor preferences involving sex, age, and tumor stage, we randomly collected 15 paired HCC tumor tissues and adjacent normal tissues. Patient information is shown in Table S2. All procedures and experiments were approved by the Biomedical Ethics Committee of the Xi'an Jiaotong University Health Science Center.

### 2.4 Western blotting

The total protein of HCC cells and HCC tissues was extracted as described previously.^20^ The mitochondrial, cytoplasmic, and nuclear fractions of HCC cells were obtained using commercial kits (Keygene, Nanjing, China). Protein samples were subjected to sodium dodecyl sulfate‐polyacrylamide gel electrophoresis and western blotting. After transferring the proteins to a membrane and blocking, the blots were sequentially incubated with primary and secondary antibodies. The bands were visualized using an enhanced ECL kit (4A Biotech, Beijing, China). Images were captured using a Tanon5200 imaging system (Tanon, Shanghai, China).

### 2.5 Plasmid transfection and construction of stable cell lines

PLAGL2‐knockdown and PLAGL2‐overexpression plasmids were prepared according to the instructions provided by OMEGA (Guangzhou, China). HCC cells were cultured for 24 h, and cultures at 50%‐70% confluence were transiently transfected with plasmids using Lipofectamine 2000 reagent for 24 h. At pre‐determined time points, the fluorescence of green fluorescent protein was observed, and the cells were collected for further experiments through screening with 1 μg/ml puromycin until all the non‐transfected control cells died. To select SMMC‐7721 cells stably overexpressing PLAGL2, 1 μg/ml puromycin was added to the cultures. Stable Bel‐7402 cell clones with high PLAGL2 expression were selected and cultured in complete RPMI‐1640 medium containing puromycin.

### 2.6 Flow cytometric analysis of cell apoptosis

HCC cells were plated in six‐well plates (4 × 10^5^ cells/well). After 24 h incubation, the cells were transfected with plasmids for 24 h or treated with SeS_2_ (0, 5, 10, and 20 μM) for 48 h. For PLAGL2 plasmid transfection, the vector plasmids were transfected and used as control groups. The cells were then treated with the apoptosis inducers CCCP (25 μM) and etoposide (40 μM) for 24 h in vector‐transfected control and PLAGL2‐overexpressing Bel‐7402 and Bel‐7404 cells to investigate the effect of PLAGL2 on apoptosis resistance. The cells were then harvested, and 5 μL Annexin V‐PE and 5 μL 7AAD staining solution were added to stain the cells. Apoptosis was detected using FACS (ACEA, Hangzhou, China).

### 2.7 Determination of mitochondrial transmembrane potential (Δψm)

Δψm was assessed using tetramethylrhodamine, ethyl ester (TMRE) staining. Briefly, after knockdown or overexpression of PLAGL2 or treatment with SeS_2_ (0, 5, 10, and 20 μM) for 48 h, the HCC cells were washed with culture medium and incubated with TMRE (100 nM, 30 min) at 37°C in the dark. The fluorescent‐labeled cells were washed again and detected via FACS (Novocyte 2040R), and the Δψm loss was quantified based on the fold change compared to the fluorescence intensity of the control.

### 2.8 Cell staining assay

Cells were plated into six‐well plates at a density of 4 × 10^5^ cells per well. For immunofluorescence assays, treated cells were fixed, blocked, and incubated with a primary antibody against p‐STAT3 (Y705) (1:200) at 37°C for 4 h. The cells were then incubated with a Cy3‐conjugated secondary antibody (1:50) at 37°C for 1 h. Nuclei were stained with DAPI. Fluorescence images were captured using an inverted fluorescence microscope (Nikon, Tokyo, Japan).

### 2.9 Cell viability assay

The effects of SeS_2_ were assessed using the MTT assay. HCC cells were seeded in 96‐well plates (2 × 10^4^ cells/well) and cultured overnight. The cells were then treated with SeS_2_ at different concentrations for 48 h. After treatment, cells were cultured with serum‐free medium and MTT (0.5 mg/ml) for 4 h, followed by the addition of 150 μL DMSO to the wells for 15 min to fully dissolve the formazan crystals. The absorbance was measured at 490 nm using a microplate reader (Bio‐Rad, Hercules, CA, USA).

### 2.10 Colony formation assay

SK‐Hep‐1 and SMMC‐7721 cells were seeded in 12‐well plates (400 cells/well) and treated with SeS_2_ (5, 10, and 20 μM) for 48 h. Subsequently, the cells were cultured in complete medium for approximately 10–15 days. After fixation, cells were stained with crystal violet. The formation of colonies on the plate and the individual colony images were acquired using a chemiluminescence and fluorescence imaging system (Sage creation, Beijing, China) and an inverted fluorescence microscope (Nikon, Tokyo, Japan), respectively.

### 2.11 RNA isolation and RT‐PCR

Total RNA from tumor tissues of patients with HCC was prepared according to the manufacturer's instructions (Vazyme, Nanjing, China). Reverse transcription and RT‐PCR were performed as described.^21^ The primer sequences for PLAGL2, MET, STAT3 and β‐actin are listed in Table S3. The relative expression of mRNA was normalized to that of β‐actin.

### 2.12 Animals and xenograft models

Four‐ to six‐week‐old (18‐22 g) BALB/c male nude mice were obtained from Shanghai SLAC Laboratory Animal Co., Ltd. and housed in the Experimental Animal Center of Xi'an Jiaotong University in a specific pathogen‐free environment. To establish the tumor xenograft model, a total of 2 × 10^6^ cells (SMMC‐7721, Ctrl/Bel‐7402, and PLAGL2/Bel‐7402 cells) were subcutaneously injected into the right armpit of mice, and the tumor was permitted to reach a volume greater than 100 mm^3^. The mice were randomly divided into two groups (*n* = 4 per group), and daily intraperitoneal administration of SeS_2_ (5 mg/kg dissolved in saline solution of 0.5% DMSO) or saline solution of 0.5% DMSO (control) was performed for 14 days. The body weights of the mice and tumor volumes were monitored daily. Tumor sizes were measured using the equation V = W^2^×L/2 mm^3^ (V, volume; W, width; L, length). The mice were sacrificed on day 14, and the tumors were removed and fixed with 4% paraformaldehyde. Tumor specimens were embedded and sectioned for use in a TUNEL assay and other immunohistochemical analyses. All procedures and experiments were approved by the Biomedical Ethics Committee of the Xi'an Jiaotong University Health Science Center.

### 2.13 TUNEL assay

The TUNEL assay was performed to investigate the presence of apoptotic cells in the xenograft tumor tissues, according to the manufacturer's instructions (Yeasen, Shanghai, China). Fluorescence images were acquired using an inverted microscope (Nikon, Tokyo, Japan).

### 2.14 Immunohistochemistry (IHC) analyses

The SV histostain kit was used according to the instructions provided by Servicebio (Wuhan, China). The antibodies used for IHC were Ki‐67 (1:2000), PLAGL2 (1:200), p‐C‐MET (1:300), p‐STAT3 (1:100), AIF (1:200), and Cleaved‐PARP (1:100). Images were acquired using a scanning microscope (Nikon, Tokyo, Japan).

### 2.15 Statistical analysis

Data are expressed as the mean ± SEM. Statistical analyses were performed using the statistical software package SPSS v.18.0. ANOVA, Dunnett's multiple comparison test, and Student's unpaired *t*‐test were performed. Significance values were set at *p < 0.05, **p < 0.01, and ***p < 0.001.

## 3 RESULTS

### 3.1 Overexpression of PLAGL2 in HCC

**FIGURE 1:**
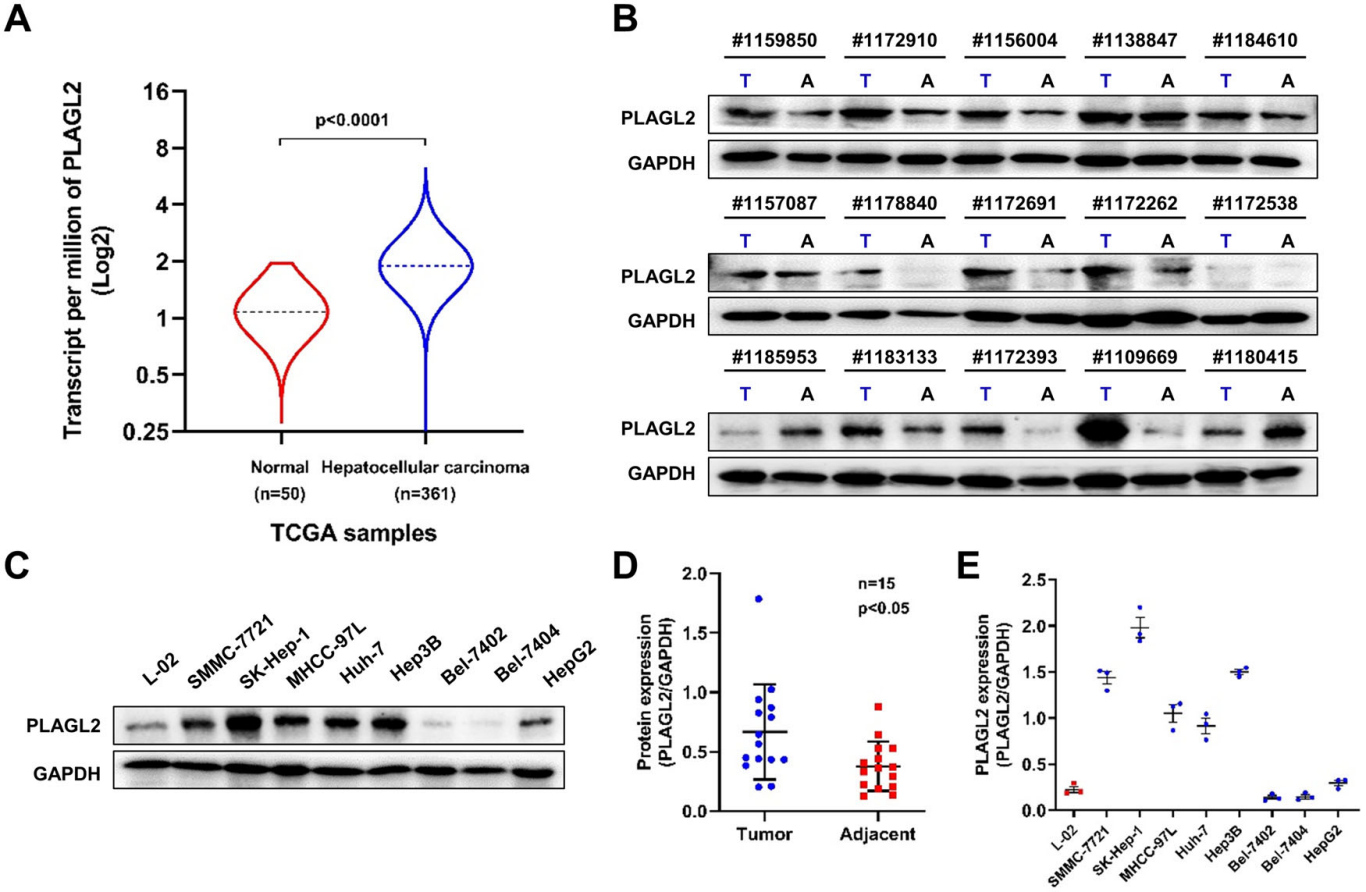
PLAGL2 expression is upregulated in human HCC tissues and cells. (A) The expression of PLAGL2 was significantly higher in the 361 HCC tissues than in the 50 adjacent normal liver tissue samples in TCGA database, as analyzed using the UALCAN web tool. (B) Protein expression levels of PLAGL2 in 15‐paired HCC tissues were examined via western blotting. (C) Protein expression levels of PLAGL2 in eight HCC cell lines and normal hepatocyte L‐02 cells. (D) Quantification of (B), the data were normalized by GAPDH. E, Quantification of (C), the data were normalized using GAPDH as the control. Data are expressed as the means ± SEM (*n* = 3). *p* value < 0.05 was considered statistically significant. Abbreviations: A, adjacent; T, tumor

The Cancer Genome Atlas (TCGA) database (Liver HCC; LIHC) analysis was performed using the UALCAN web tool.^22^ The results showed that PLAGL2 was overexpressed in HCC tumor samples compared to that in normal samples (Figures 1A and S1A). We further analyzed the expression of PLAGL2 in 15 paired HCC and non‐carcinoma tissues via western blot analysis. In this cohort, we found that PLAGL2 expression was considerably upregulated in HCC tumor tissues compared to that in adjacent non‐tumor tissues (Figure 1B,D). Western blotting was performed to investigate PLAGL2 protein expression in eight HCC cell lines and normal hepatocyte L‐02 cells. The protein expression of PLAGL2 was higher in the five tested HCC cells than in the normal L‐02 cells (Figure 1C,E). Therefore, these data imply that PLAGL2 expression is correlated with HCC progression.

### 3.2 PLAGL2 contributes to mitochondrial apoptosis resistance in HCC cells

**FIGURE 2:**
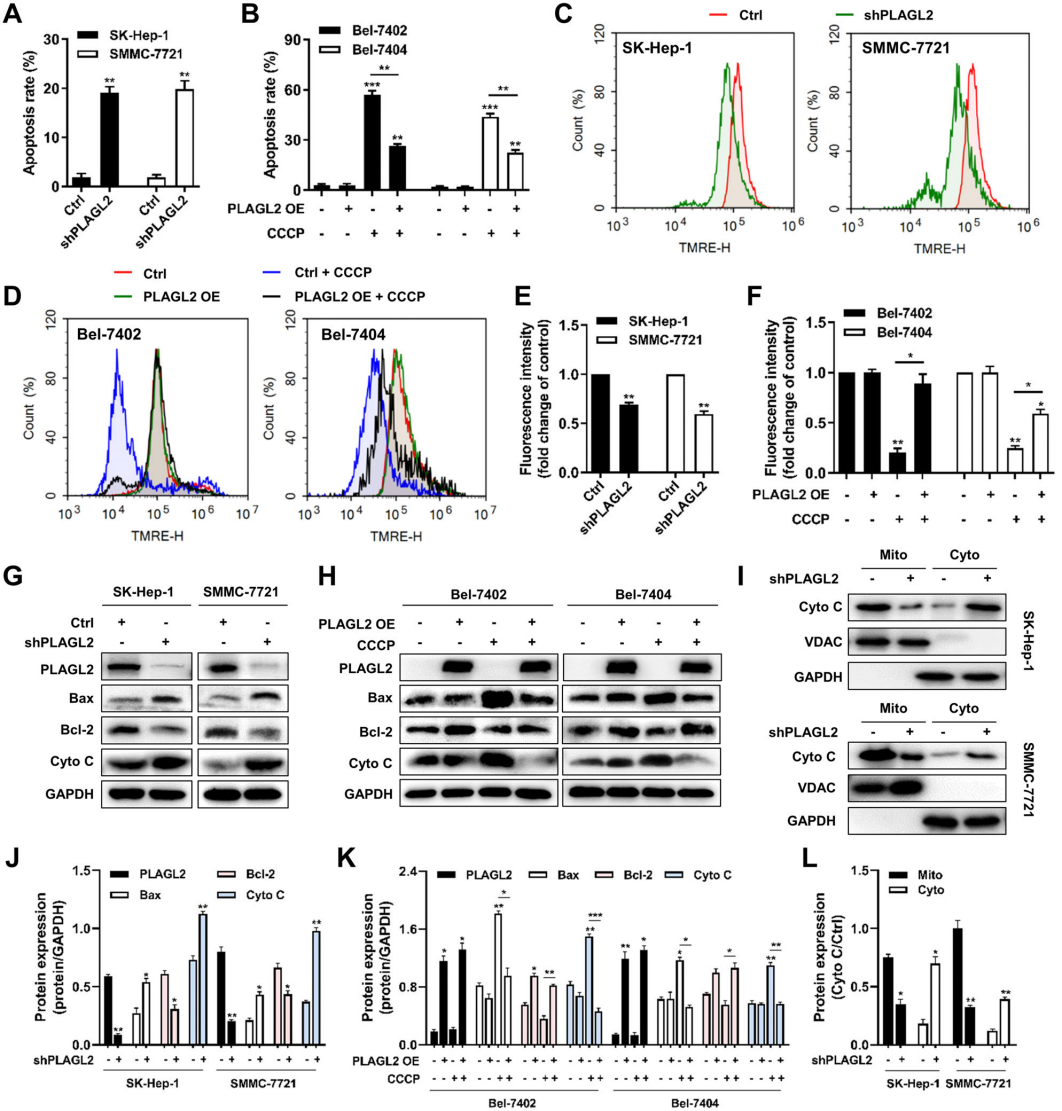
PLAGL2 promotes the resistance of CCCP‐induced HCC cells apoptosis. (A) Knockdown of endogenous PLAGL2 expression in SK‐Hep‐1 and SMMC‐7721 cells induced cell apoptosis. Cell apoptosis was measured via Annexin V‐PE/7AAD staining in wild‐type control (Ctrl) and PLAGL2‐knockdown (shPLAGL2) SK‐Hep‐1 and SMMC‐7721 cells. The percentage of apoptotic cells was indicated. The results shown were representative of three independent experiments. (B) Overexpression of PLAGL2 in Bel‐7402 and Bel‐7404 cells promoted resistance of CCCP‐induced cells apoptosis. Wild‐type control (Ctrl) or PLAGL‐overexpressing (PLAGL2 OE) Bel‐7402 and Bel‐7404 cells were treated with 25 μM CCCP for 24 h. (C) Knockdown of endogenous PLAGL2 expression in SK‐Hep‐1 and SMMC‐7721 cells induced the loss of mitochondrial membrane potential (Δψm). Δψm was assessed through the retention of the dye TMRE using flow cytometry. (D) Overexpression of PLAGL2 in Bel‐7402 and Bel‐7404 cells restored the normal Δψm values in the presence of CCCP. E, Quantification histogram of (C) based on TMRE fluorescence intensity (fold change of control). (F) Quantification histogram of (D) based on TMRE fluorescence intensity (fold change of control). (G) Western blotting analysis for Bax, Bcl‐2, and Cyto C expression in the Ctrl/SK‐Hep‐1, shPLAGL2/SK‐Hep‐1, Ctrl/SMMC‐7721, and shPLAGL2/SMMC‐7721 cells. (H) Western blotting analysis for Bax, Bcl‐2 and Cyto C expression in the CCCP‐treated Ctrl/Bel‐7402, PLAGL2/Bel‐7402, Ctrl/Bel‐7404, and PLAGL2/Bel‐7404 cells. (I) Cell lysates of Ctrl/SK‐Hep‐1, shPLAGL2/SK‐Hep‐1, Ctrl/SMMC‐7721, and shPLAGL2/SMMC‐7721 cells were divided into mitochondrial and cytoplasmic fractions. Cyto C levels were measured by western blotting. GAPDH and VDAC served as controls. (J) Bar plot of (G.) (K) Bar plot of (H). (L) Bar plot of (I). Data are expressed as the means ± SEM (*n* = 3). *p < 0.05, **p < 0.01, ***p < 0.001 compared with the control group

It has been reported that PLAGL2 enhances HCC cell proliferation and metastasis in vitro and in vivo, but the functional role and underlying mechanism of action of PLAGL2 in HCC remain poorly understood.^9^ Based on the escape from programmed apoptosis and the overactivation of PLAGL2 in HCC, we speculated that PLAGL2 also plays a crucial role in HCC apoptotic resistance. To investigate the oncogenic role of PLAGL2 in HCC, PLAGL2‐knockdown and PLAGL2‐overexpressing cell lines were constructed. Based on the results shown in Figure 1C, we utilized the representative PLAGL2‐overexpressing SMMC‐7721 and SK‐Hep‐1 cells as model cells to knock down PLAGL2, while PLAGL2‐deficient Bel‐7402 and Bel‐7404 cells were used to overexpress PLAGL2. In this study, we investigated the effects of PLAGL2 on apoptosis. The results showed that PLAGL2 knockdown induced apoptosis in SK‐Hep‐1 and SMMC‐7721 cells, unlike in vector‐transfected control cells (Figures 2A and S2A). Moreover, forced PLAGL2 expression in Bel‐7402 and Bel‐7404 cells reduced the effect of the apoptosis inducer CCCP compared to that in vector‐transfected cells (Figures 2B and S2B). Consistent with the cell apoptosis results, Δψm as assessed via TMRE staining was reduced in PLAGL2‐knockdown SK‐Hep‐1 and SMMC‐7721 cells (Figure 2C,E). However, overexpression of PLAGL2 in Bel‐7402 and Bel‐7404 cells significantly rescued the CCCP‐mediated Δψm loss (Figure 2D,F). Furthermore, the results of western blotting analysis showed that PLAGL2 knockdown upregulated Bax expression and increased the release of Cyto C from the mitochondria to the cytoplasm, but downregulated Bcl‐2 expression (Figure 2G,I,J,L), whereas PLAGL2 overexpression exhibited the opposite effect, which reversed CCCP‐induced Bax activation, Cyto C release, and Bcl‐2 inhibition (Figure 2H,K). In addition, PLAGL2 overexpression reversed etoposide‐induced apoptosis in Bel‐7402 and Bel‐7404 cells (Figure S2C and D). Meanwhile, we also performed both overexpression and silencing of PLAGL2 in the same MHCC‐97L cell line, and investigated the role of PLAGL2 in MHCC‐97L cell apoptosis resistance. As shown in Figure S3, overexpression of PLAGL2 may lead to apoptosis resistance in MHCC‐97L cells. These results indicated that PLAGL2 enhanced HCC cell apoptosis resistance in vitro.

### 3.3 The C‐MET/STAT3 signaling axis is a novel downstream target of PLAGL2

**FIGURE 3:**
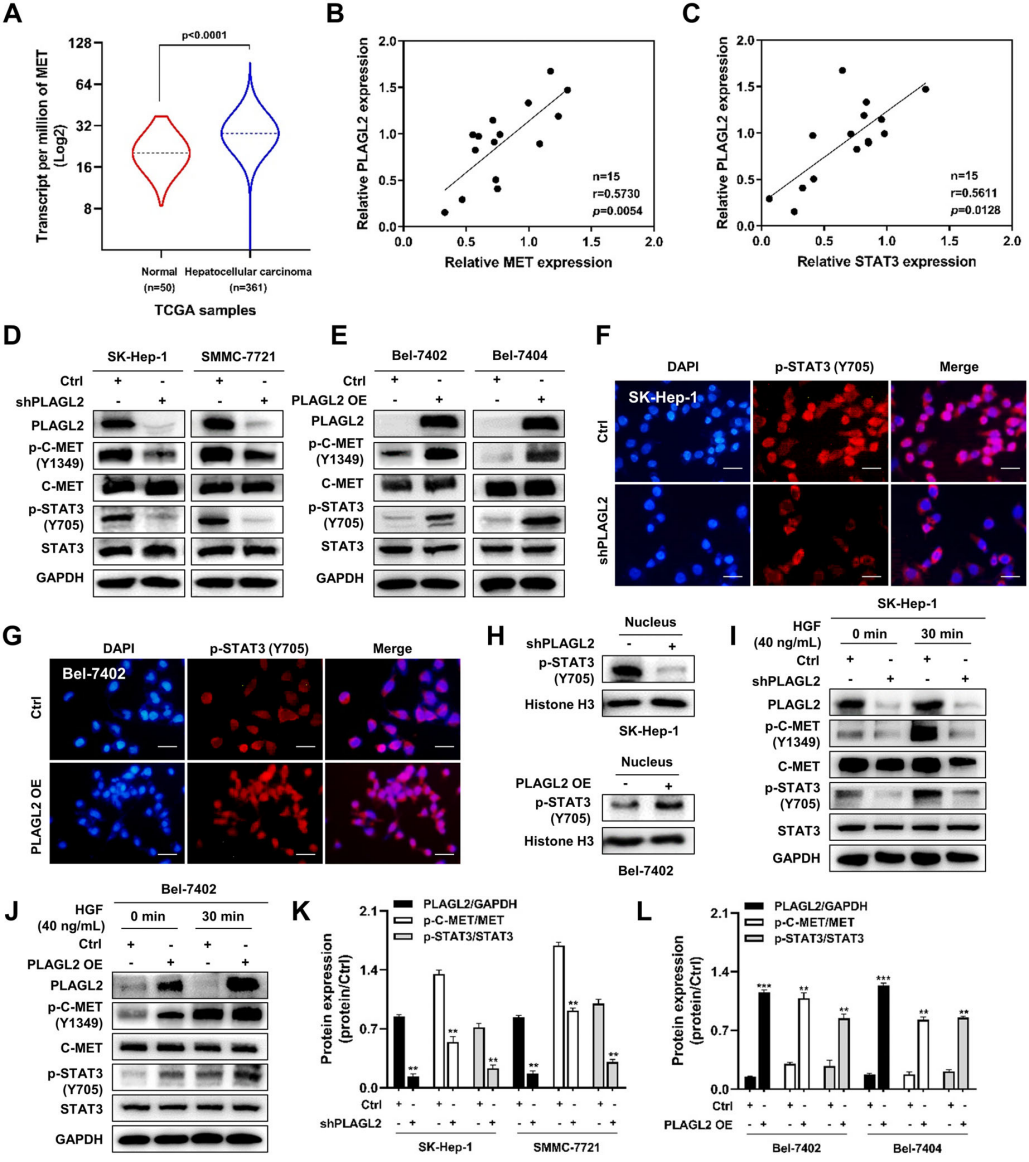
PLAGL2 regulates the C‐MET/STAT3 signaling axis. (A) The expression of MET was significantly higher in the 361 HCC tissues than in the 50 adjacent normal liver tissue samples in TCGA database as analyzed using the UALCAN web tool. (B) The positive correlation between the mRNA expression of PLAGL2 and MET in 15 paired HCC tumor tissues. (C) The positive correlation between the mRNA expression of PLAGL2 and STAT3 in 15 paired HCC tumor tissues. (D) C‐MET and STAT3 expression levels following PLAGL2‐knockdown in the two HCC cell lines (Ctrl/SK‐Hep‐1, shPLAGL2/SK‐Hep‐1, Ctrl/SMMC‐7721, and shPLAGL2/SMMC‐7721) were detected at the total and phosphorylated protein levels via western blotting. (E) Total and phosphorylated C‐MET and STAT3 protein levels following PLAGL2‐overexpression in two HCC cell lines (Ctrl/Bel‐7402, PLAGL2/Bel‐7402, Ctrl/Bel‐7404, and PLAGL2/Bel‐7404). The nuclear translocation of p‐STAT3 (Y705) was impeded in PLAGL2‐knockdown SK‐Hep‐1 cells (F) but was markedly promoted in PLAGL2‐overexpressing Bel‐7402 cells (G) as revealed via immunofluorescence microscopy (the scale bar represents 100 μm). (H) Nuclear fractions of Ctrl/SK‐Hep‐1, shPLAGL2/SK‐Hep‐1, Ctrl/SMMC‐7721, and shPLAGL2/SMMC‐7721 cells were extracted. p‐STAT3 (Y705) levels were measured via western blotting. Histone H3 served as control. I, Ctrl/SK‐Hep‐1 and shPLAGL2/SK‐Hep‐1 cells were cultured in serum‐free MEM for 24 h, and then exposed to HGF (40 ng/ml) for 30 min, followed by western blotting with primary antibodies against PLAGL2, p‐C‐MET (Y1349), C‐MET, p‐STAT3 (Y705), and STAT3. (J) Ctrl/Bel‐7402 and PLAGL2/Bel‐7402 cells were cultured in serum‐free RPMI‐1640 for 24 h, and then exposed to HGF (40 ng/ml) for 30 min, followed by western blotting with the indicated primary antibodies. (K) Bar plot of (D). L, Bar plot of (E). Data are expressed as the means ± SEM (*n* = 3). **p < 0.01, ***p < 0.001 compared with the control group

C‐MET is known to be overexpressed in HCC, and the HGF/C‐MET axis is involved in cell proliferation, angiogenesis, and apoptosis by activating multiple downstream signaling pathways, such as STAT3 and MAPK.^13^, ^22^, ^23^ To confirm the relevance of PLAGL2 and C‐MET in HCC progression, we analyzed the mRNA data of TCGA liver cancer cohorts (LIHC) using the UALCAN web tool.^24^ We found that MET expression was significantly higher in HCC tumor tissues than in 50 normal tissue samples (Figures 3A and S1B), and a positive correlation between the expression of PLAGL2 and MET was observed in 15‐paried HCC tumor tissues (Figure 3B) (*r* = 0.5730, *p* < 0.01). Moreover, a positive correlation between the expression of both PLAGL2 and STAT3 was observed in the same cohort (Figure 3C), and STAT3 is a vital target gene of C‐MET (*r* = 0.5611, *p* < 0.05). Next, the phosphorylation of C‐MET and STAT3 was evaluated in PLAGL2‐knockdown and PLAGL2‐overexpressing HCC cells. As shown in Figure 3D,E, silencing of PLAGL2 in SK‐Hep‐1 and SMMC‐7721 cells induced the arrest of C‐MET and STAT3, whereas the co‐occurrence of PLAGL2, p‐C‐MET (Y1349), and p‐STAT3 (Y705) overexpression was found in constructed Bel‐7402 and Bel‐7404 cells compared to that in vector‐transfected cells (Figure 3K,L). The regulation of C‐MET/STAT3 signaling by PLAGL2 was also confirmed in MHCC‐97L cells (Figure S3 D,E). Furthermore, Figure 3F,G indicated that the red fluorescence in the nucleus was significantly decreased in shPLAGL2/SK‐Hep‐1 cells, and that PLAGL2 overexpression markedly promoted the nuclear translocation of p‐STAT3 in Bel‐7402 cells (Figure S4A). The detection of p‐STAT3 expression in the nuclear fraction also confirmed that PLAGL2 contributed to the nuclear distribution of p‐STAT3 (Figures 3H and S4B). Moreover, HGF‐stimulated C‐MET activation and downstream STAT3 signaling pathway were investigated. C‐MET phosphorylation (p‐C‐MET Y1349) and STAT3 phosphorylation (p‐STAT3 Y705) were markedly reduced in PLAGL2‐knockdown cells compared to that in HGF‐stimulated Ctrl/SK‐Hep‐1 cells (Figures 3I and S4C). In addition, there was an obvious increase in p‐C‐MET and p‐STAT3 levels in PLAGL2‐overexpressing Bel‐7402 cells compared to those in HGF‐stimulated Ctrl/Bel‐7402 cells (Figures 3J and S4D), indicating that C‐MET‐regulated signaling contributed to PLAGL2‐induced HCC apoptosis resistance.

### 3.4 Selenium sulfide induces the growth inhibition and mitochondrial apoptosis of HCC cells in vitro and in vivo

**FIGURE 4:**
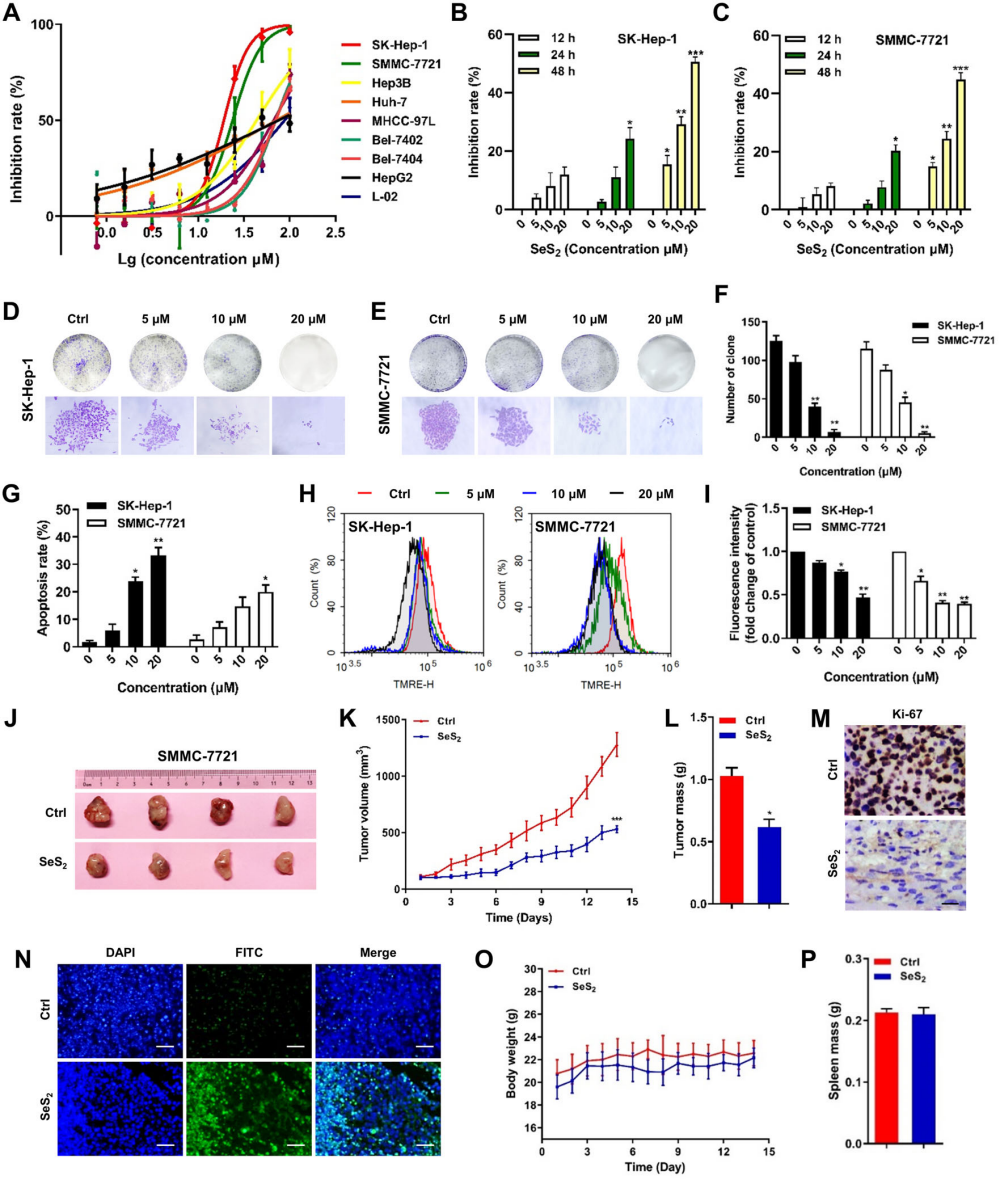
Selenium sulfide inhibits the proliferation and induces the mitochondrial apoptosis of HCC cells in vitro and in vivo. (A) Effect of SeS_2_ on the proliferation of SK‐Hep‐1, SMMC‐7721, MHCC‐97L, Huh‐7, Hep3B, Bel‐7402, Bel‐7404, HepG2, and normal hepatocyte L‐02 cells. Cells were treated with SeS_2_ (0.78125‐100 μM) for 48 h, and cytotoxicity was analyzed using the MTT assay. SK‐Hep‐1 (B) and SMMC‐7721 (C) cells were treated with 5, 10, and 20 μM SeS_2_ for 12, 24, 48 h and cell viability were assessed using the MTT assay. The effects of SeS_2_ on the colony formation of SK‐Hep‐1 cells (D) and SMMC‐7721 cells (E). The colony formation (upper line) and the individual colonies (lower line) were photographed (200 × magnification). (F) Quantification histogram of (D) and (E). (G) Effects of SeS_2_ on the apoptosis of SK‐Hep‐1 and SMMC‐7721 cells. The apoptosis ratio was measured via flow cytometry analysis using Annexin V‐PE/7AAD double staining. (H) Effects of SeS_2_ on the Δψm. Δψm was assessed through the retention of the dye TMRE using flow cytometry. (I) Quantification histogram of (H) based on TMRE fluorescence intensity (fold change of control). (J) Images of tumors excised from four nude mice at 14 days after intraperitoneal injection of saline solution or SeS_2_ (5 mg/kg) in SMMC‐7721 xenograft tumors. (K) Volume changes of SMMC‐7721 tumors were measured every day. (L) Effects of SeS_2_ treatment on the masses of SMMC‐7721 tumors. (M) Representative images of the immunohistochemical analysis of Ki‐67 expression in SMMC‐7721 xenograft samples. The scale bar represents 50 μm. (N) Representative images of the TUNEL assay in SMMC‐7721 xenograft samples. The scale bar represents 200 μm. (O) Body weight changes in mice harboring SMMC‐7721 tumors. (P) Spleen mass of mice harboring SMMC‐7721 xenograft tumors. Data are expressed as the means ± SEM (*n* = 4). *p < 0.05, **p < 0.01, ***p < 0.001 compared with the control group

The MTT assay was conducted to test the effects of SeS_2_ on the proliferation of HCC cell lines. As shown in Figure 4A, SeS_2_ had a more significant anti‐proliferative effect in SK‐Hep‐1 and SMMC‐7721 cells than in other HCC cells after 48 h of treatment, and it was found to be relatively non‐toxic to normal hepatocyte L‐02 cells (Table S4). Furthermore, SeS_2_ inhibited the growth of SK‐Hep‐1 and SMMC‐7721 cells in a time‐dependent manner (Figure 4B,C). We also investigated the effect of SeS_2_ on colony formation. The results indicated that SeS_2_ markedly inhibited cell colony formation in SK‐Hep‐1 and SMMC‐7721 cells at concentrations of 10 μM and 20 μM (Figure 4D‐F). The apoptosis induction capability of SeS_2_ was demonstrated by Annexin V‐PE/7AAD staining in SK‐Hep‐1 and SMMC‐7721 cells (Figures 4G and S5A). Furthermore, the Δψm of SeS_2_‐treated SK‐Hep‐1 and SMMC‐7721 cells was significantly decreased, as shown by the TMRE staining results (Figure 4H,I).

Furthermore, the antitumor effects of SeS_2_ were evaluated in SMMC‐7721 xenografts in nude mice. Treatment with SeS_2_ (5 mg/kg) significantly delayed tumor growth, decreased tumor volume, and tumor mass (Figure 4J,K,L). Reduced expression of Ki‐67 in SMMC‐7721 tumor tissues was also observed after SeS_2_ treatment (Figures 4 M and S5B). In addition, SeS_2_ increased the number of apoptotic cells in SMMC‐7721 xenograft tumors, further indicating the in vivo apoptosis‐inducing function of SeS_2_ (Figures 4N and S5C). Nevertheless, no obvious loss of body weight or spleen mass was observed in the mice during the experimental period (Figure 4O,P).

### 3.5 PLAGL2 serves as a promising target for selenium sulfide, and its overexpression sensitizes the effect of selenium sulfide in HCC cells in vitro and in vivo

**FIGURE 5:**
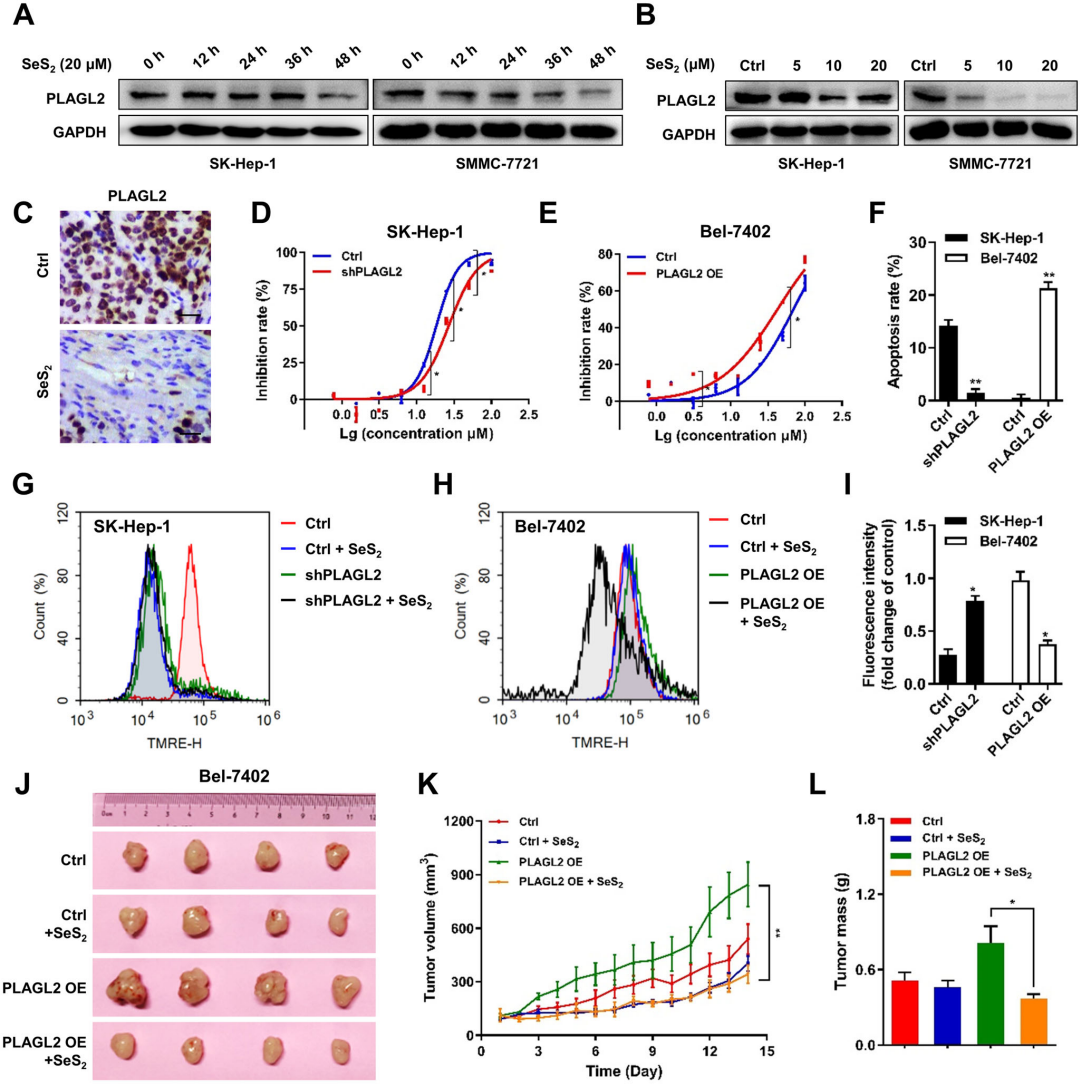
PLAGL2 serves as a promising target for selenium sulfide induced proliferation inhibition and apoptosis of HCC cells in vitro and in vivo. (A) Protein levels of PLAGL2 in SK‐Hep‐1 and SMMC‐7721 cells exposed to SeS_2_ (20 μM) for different durations. (B) Protein expression of PLAGL2 in SK‐Hep‐1 and SMMC‐7721 cells treated with SeS_2_ for 48 h. (C) Representative images of the immunohistochemical analysis of PLAGL2 expression in SMMC‐7721 xenograft samples. The scale bar represents 50 μm. (D) Effects of SeS_2_ on the proliferation of Ctrl/SK‐Hep‐1 and shPLAGL2/SK‐Hep‐1 cells. (E) Effects of SeS_2_ on the proliferation of Ctrl/Bel‐7402 and PLAGL2/Bel‐7402 cells. (F) Effects of SeS_2_ on the apoptosis of Ctrl/SK‐Hep‐1, shPLAGL2/SK‐Hep‐1, Ctrl/Bel‐7402, and PLAGL2/Bel‐7402 cells. (G) Effects of SeS_2_ on the Δψm of Ctrl/SK‐Hep‐1 and shPLAGL2/SK‐Hep‐1 cells. (H) Effects of SeS_2_ on the Δψm of Ctrl/Bel‐7402 and PLAGL2/Bel‐7402 cells. (I) Quantification histogram of (G) and (H) based on TMRE fluorescence intensity (fold change of control). (J) Images of tumors excised from four nude mice at 14 days after intraperitoneal injection of saline solution or SeS_2_ (5 mg/kg) in Ctrl/Bel‐7402 and PLAGL2/Bel‐7402 xenograft tumors. (K) Tumor volume changes in the mice were measured every day. (L) Effects of SeS_2_ on the masses of Ctrl/Bel‐7402 and PLAGL2/Bel‐7402 xenograft tumors. Data are expressed as the means ± SEM (*n* = 4). *p < 0.05, **p < 0.01 compared with the control group

Interestingly, we found that the effect of SeS_2_ was positively correlated with the protein levels of PLAGL2. To reveal the importance of PLAGL2 in the anti‐HCC effect of SeS_2_, we measured PLAGL2 protein levels in SK‐Hep‐1 and SMMC‐7721 cells after treatment with SeS_2_ at different concentrations for 48 h or at 20 μM for different durations. Figure 5A indicated that PLAGL2 expression was downregulated after 48 h of SeS_2_ exposure in SK‐Hep‐1 cells, but it was notably decreased after 24 h of SeS_2_ exposure in SMMC‐7721 cells (Figure S6A). Figure 5B showed that PLAGL2 protein levels in SK‐Hep‐1 and SMMC‐7721 cells decreased in a concentration‐dependent manner after treatment with SeS_2_ for 48 h (Figure S6B). SeS_2_ treatment also downregulated PLAGL2 levels in SMMC‐7721 tumor tissues (Figures 5C, and S6C). PLAGL2‐knockdown SK‐Hep‐1 and PLAGL2‐overexpressing Bel‐7402 cells were utilized to confirm the role of PLAGL2 in SeS_2_‐meidiated cell proliferation inhibition and apoptosis induction in HCC. Figure S6D showed that SeS_2_ could significantly downregulate PLAGL2 protein expression in Ctrl/SK‐Hep‐1 and PLAGL2/Bel‐7402 cells. The results also showed that PLAGL2 knockdown decreased the inhibitory effect of SK‐Hep‐1 cells on SeS_2_, while overexpression of PLAGL2 increased the sensitivity of SeS_2_ to Bel‐7402 cells (Figure 5D,E). Additionally, flow cytometric analysis showed that PLAGL2 knockdown interfered with SeS_2_‐induced apoptosis and decreased Δψm in SK‐Hep‐1 cells, whereas PLAGL2 overexpression increased the apoptosis ratio and Δψm loss induced by SeS_2_ in Bel‐7402 cells (Figures 5F‐I and S6E,F).

Next, we performed tumorigenesis assays in vivo. PLAGL2‐overexpressing Bel‐7402 cells were injected into the right armpit of mice. Overexpression of PLAGL2 in Bel‐7402 cells significantly stimulated tumor growth compared to that in vector‐transfected cells and facilitated the inhibitory effect of SeS_2_ on tumor growth (Figure 5J), which was presented as the tumor volume and the final change in xenograft tumor weight (Figure 5K,L). Moreover, no obvious reduction in body weight or spleen mass was observed (Figure S7). These findings confirmed that SeS_2_ inhibited cell proliferation and induced apoptosis by downregulating PLAGL2 expression.

### 3.6 Selenium sulfide inhibits C‐MET and downstream STAT3, AKT/mTOR and MAPK signaling

**FIGURE 6:**
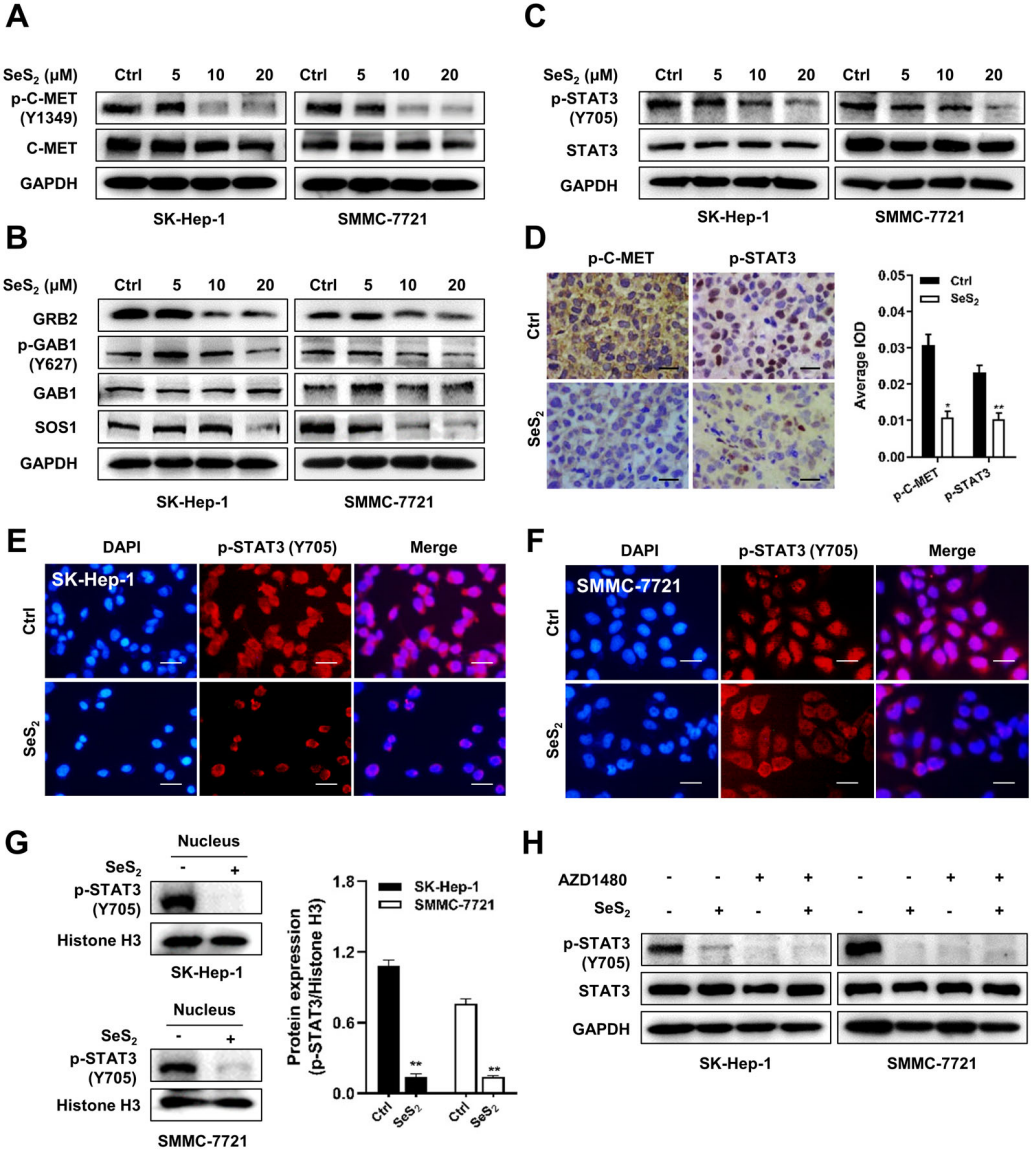
Selenium sulfide inhibits the C‐MET/STAT3 signaling axis. (A) Protein expression of p‐C‐MET (Y1349) and C‐MET in SK‐Hep‐1 and SMMC‐7721 cells treated with SeS_2_ for 48 h. (B) Protein expression of GRB2, p‐GAB1 (Y627), GAB1, and SOS1 in SK‐Hep‐1 and SMMC‐7721 cells treated with SeS_2_ for 48 h. (C) Protein expression of p‐STAT3 (Y705) and STAT3 in SK‐Hep‐1 and SMMC‐7721 cells treated with SeS_2_ for 48 h. (D) Representative images of the immunohistochemical analysis of p‐C‐MET and p‐STAT3 expression in SMMC‐7721 xenograft samples, the scale bar represents 50 μm. Representative images of the immunofluorescence analysis of p‐STAT3 (Y705) protein in SK‐Hep‐1 (E) and SMMC‐7721 (F) cells treated with SeS_2_ (20 μM) for 48 h. p‐STAT3 (Y705) (red), DAPI (blue) staining, and merged images indicated the nuclear translocation and expression of p‐STAT3. The scale bar represents 100 μm. (G) Nuclear fractions of Ctrl and SeS_2_‐treated (20 μM) SK‐Hep‐1 and SMMC‐7721 cells were extracted. p‐STAT3 (Y705) levels were measured via western blotting. Histone H3 served as control. H, Protein expression of p‐STAT3 (Y705) and STAT3 in SK‐Hep‐1 and SMMC‐7721 cells after treatment with 20 μM SeS_2_ for 48 h in the presence of 3 μM AZD1480 for 12 h. Data are expressed as the means ± SEM (n = 3). *p < 0.05, **p < 0.01 compared with the control group

Based on the previously revealed relevance of PLAGL2 and C‐MET in HCC, we further investigated the effect of SeS_2_ on the C‐MET/STAT3 axis and typical downstream AKT/mTOR and MAPK pathways. A significant decrease in C‐MET phosphorylation levels was observed in SK‐Hep‐1 and SMMC‐7721 cells after treatment with 10 μM and 20 μM SeS_2_ (Figures 6A and S8A). The protein expression of associated adapter proteins, including GRB2, p‐GAB1, and SOS1, was also decreased by SeS_2_ at concentrations of 10 μM and 20 μM (Figures 6B and S8B,C). Meanwhile, SeS_2_ treatment decreased the phosphorylation of STAT3 at the tyrosine 705 site in SMMC‐7721 cells in a dose‐dependent manner but inhibited STAT3 phosphorylation of SK‐Hep‐1 cells at a concentration of 20 μM (Figures 6C and S8A). Treatment with SeS_2_ also decreased p‐C‐MET and p‐STAT3 expression in SMMC‐7721 tumor tissues (Figure 6D). The weak red fluorescence observed in the immunostaining of SK‐Hep‐1 and SMMC‐7721 cells indicated that p‐STAT3 (Y705) expression was decreased, and its distribution pattern shifted from being present cell‐wide to being isolated in the cytoplasm after SeS_2_ treatment, suggesting that SeS_2_ impeded the nuclear translocation of p‐STAT3 (Y705) (Figures 6E,F and S8D). Moreover, SeS_2_ inhibited the nuclear expression of p‐STAT3 (Y705) (Figure 6G). Co‐treatment with SeS_2_ and the STAT3 inhibitor AZD1480 significantly suppressed STAT3 phosphorylation (Figures 6H and S8E).

**FIGURE 7:**
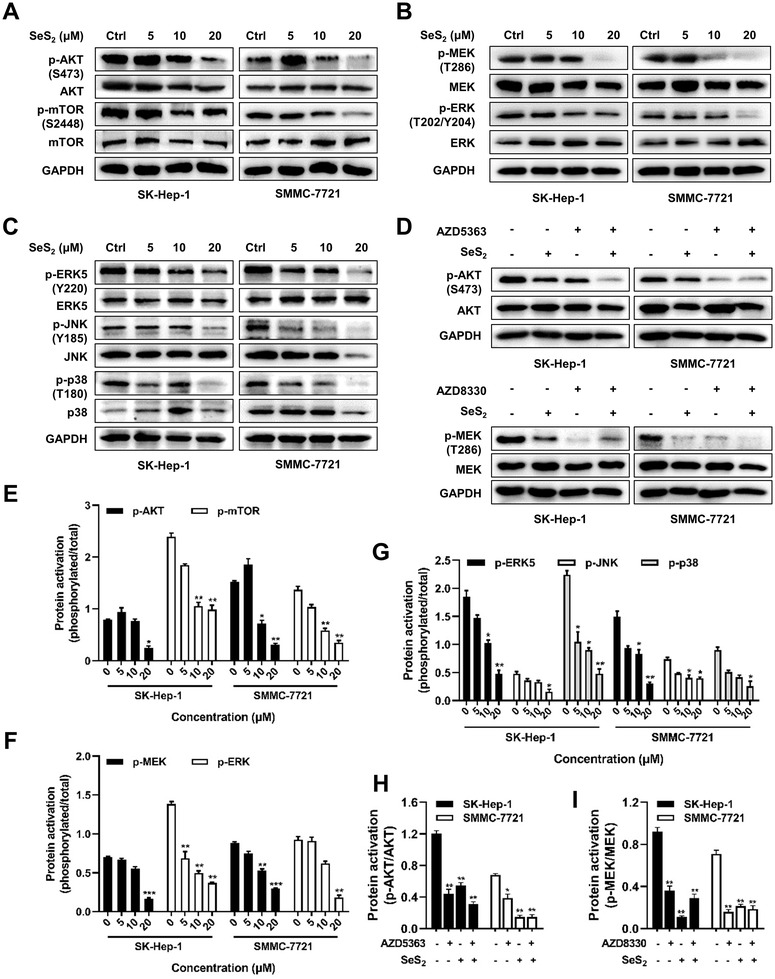
Selenium sulfide suppresses C‐MET downstream in the AKT/mTOR and MAPK signaling pathways. (A) Protein expression of p‐AKT (S473), AKT, p‐mTOR (S2448), and mTOR in SK‐Hep‐1 and SMMC‐7721 cells treated with SeS_2_ for 48 h at indicated concentrations. (B) Protein expression of p‐MEK (T286), MEK, p‐ERK (T202/Y204), and ERK in SK‐Hep‐1 and SMMC‐7721 cells treated with SeS_2_ for 48 h. (C) Protein expression of p‐ERK5 (Y220), ERK5, p‐JNK (Y185), JNK, p‐p38 (T180), and p38 in SK‐Hep‐1 and SMMC‐7721 cells treated with SeS_2_ for 48 h. (D) Protein expression of p‐AKT (S473), AKT, p‐MEK (T286), and MEK in SK‐Hep‐1 and SMMC‐7721 cells after treatment with 20 μM SeS_2_ in the presence of 1 μM AZD5363 for 12 h or 0.5 μM AZD8330 for 6 h. E, Bar plot of (A). (F) Bar plot of (B). (G) Bar plot of (C). (H and I) Bar plot of (D). Data are expressed as the means ± SEM (*n* = 3). *p < 0.05, **p < 0.01, ***p < 0.001 compared with the control group

In addition, the activation of AKT and mTOR in SK‐Hep‐1 and SMMC‐7721 cells was inhibited by SeS_2_ at a concentration of 20 μM (Figure 7A,E). Figure 7B,C showed that SeS_2_ downregulated the MAPK signaling pathway involving MEK, ERK, ERK5, JNK, and p38 at different levels (Figure 7F,G). To further verify the effect of SeS_2_ on AKT/mTOR and MAPK signaling, the AKT inhibitor AZD5363 and MEK inhibitor AZD8330 were used. As shown in Figure 7D, the combination of SeS_2_ and AZD5363 synergistically blocked AKT activation in SK‐Hep‐1 cells (Figure 7H), but SeS_2_ and AZD5363 competitively inhibited the phosphorylation of MEK (Figure 7I).

### 3.7 Selenium sulfide triggers intrinsic mitochondrial apoptosis

**FIGURE 8:**
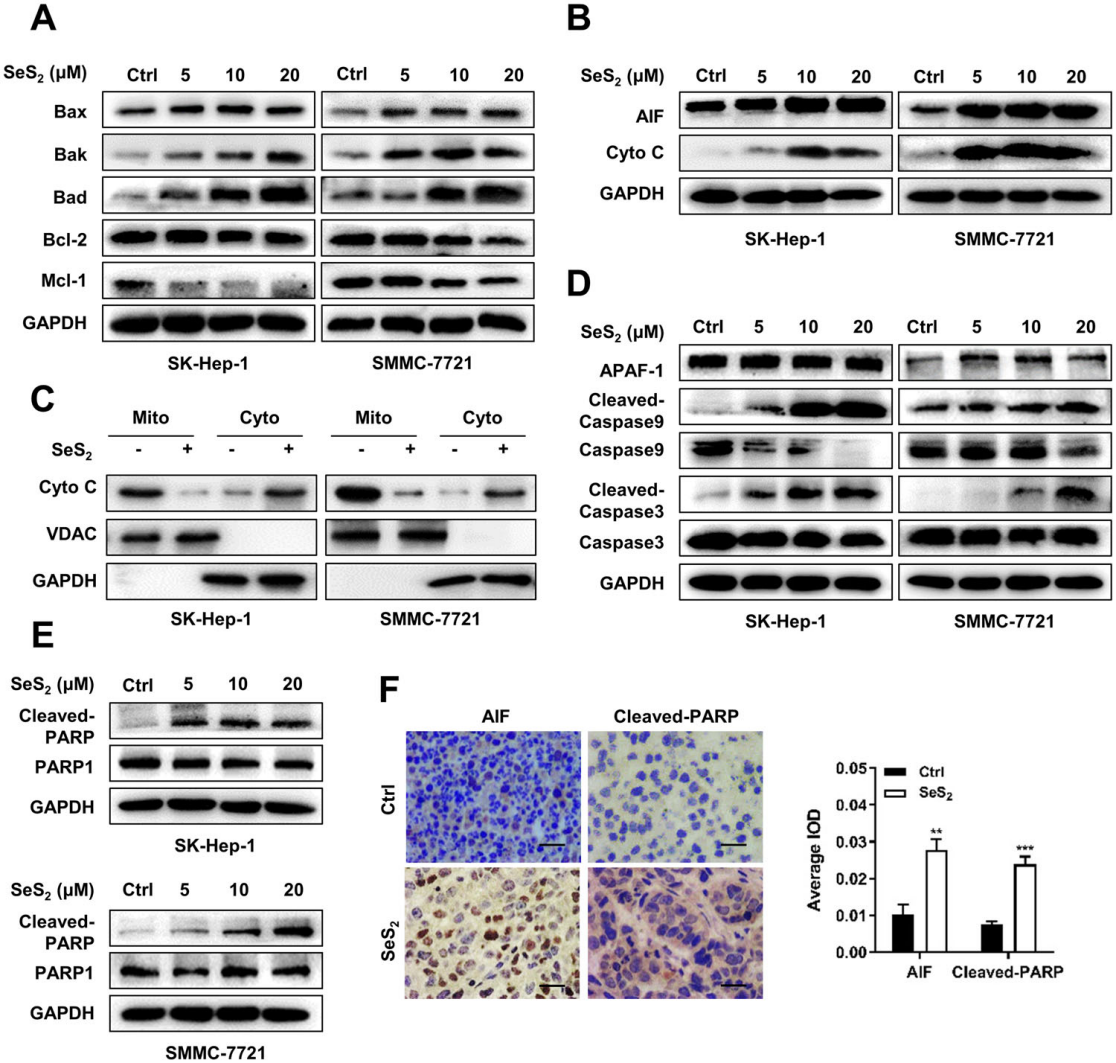
Selenium sulfide regulates the mitochondrial apoptosis family of proteins and triggers the downstream Caspase cascade. (A) Protein expression of Bax, Bak, Bad, Bcl‐2, and Mcl‐1 in SK‐Hep‐1 and SMMC‐7721 cells treated with SeS_2_ for 48 h. (B) Protein expression of AIF and Cyto C in SK‐Hep‐1 and SMMC‐7721 cells treated with SeS_2_ for 48 h. (C) Cell lysates of Ctrl and SeS_2_‐treated (20 μM) SK‐Hep‐1 and SMMC‐7721 cells were divided into mitochondrial and cytoplasmic fractions. Cyto C levels were measured via western blotting. GAPDH and VDAC served as controls. (D) Protein expression of APAF‐1, Cleaved‐Caspase9, Caspase9, Cleaved‐Caspase3, and Caspase3 in SK‐Hep‐1 and SMMC‐7721 cells treated with SeS_2_ (20 μM) for 48 h. (E) Protein expression of Cleaved‐PARP and PARP1 in SK‐Hep‐1 and SMMC‐7721 cells treated with SeS_2_ (20 μM) for 48 h. (F) Representative images and quantification of the immunohistochemical analysis of AIF and Cleaved‐PARP expression in SMMC‐7721 xenograft samples. The scale bar represents 50 μm. Data are expressed as the means ± SEM (*n* = 3). **p < 0.01, ***p < 0.001 compared with the control group

To explore the mechanism underlying SeS_2_‐induced mitochondrial apoptosis, the intrinsic apoptotic pathway was investigated. The upregulation of pro‐apoptotic proteins (Bax, Bak, and Bad), as well as the downregulation of anti‐apoptotic proteins (Bcl‐2 and Mcl‐1), indicated that SeS_2_ induced apoptosis in SK‐Hep‐1 and SMMC‐7721 cells through mitochondrial pathways (Figures 8A and S9A,B). Furthermore, the levels of apoptosis‐inducing factor (AIF), Caspase‐9, Caspase‐3, and PARP cleavage and Cyto C release increased in a dose‐dependent manner in these two cells after SeS_2_ treatment (Figures 8B,D,E, and S9). The release of Cyto C from the mitochondria to the cytoplasm in SeS_2_‐treated SK‐Hep‐1 and SMMC‐7721 cells was further confirmed by western blot analysis (Figure 8C). Additionally, the expression levels of AIF and Cleaved‐PARP were inspected in vivo via immunohistochemical staining. Figure 8F showed that the levels of AIF and Cleaved‐PARP were notably increased in SeS_2_‐treated SMMC‐7721 tumor tissues.

## 4 DISCUSSION

Failure and blockage of apoptosis are hallmarks of HCC tumorigenesis. For a long time, the mitochondria‐mediated “intrinsic” apoptosis pathway has been considered as the main form of programmed cell death and is the basis for developing anti‐cancer drugs.^25^ However, since cancer cells can eventually acquire resistance against apoptosis, current treatment outcomes are usually far from satisfactory.^26^ Therefore, the study of tumor apoptosis resistance is essential for the development of new therapeutic targets for HCC. In this study, PLAGL2 expression was found to be upregulated in HCC tissues and in the majority of HCC cells. Emerging evidence has revealed that PLAGL2 acts as an oncogene in various cancers, such as neuroblastoma, non‐small cell lung cancer, prostate cancer, colorectal cancer, and leukemia.^27^, ^28^, ^29^, ^30^ However, its biological role in HCC remains unclear. This study revealed that PLAGL2 mediated the apoptosis resistance of HCC by activating the C‐MET/STAT3 signaling axis and that SeS_2_, a potential PLAGL2 inhibitor, exerted prominent anti‐proliferative and apoptosis‐inductive effects in HCC both in vitro and in vivo.

PLAGL2, a close homolog of PLAG1, was proposed to participate in the physiological regulation of different types of cells, including HCC cells.^31^ In our study, we found that CCCP and etoposide‐induced mitochondrial apoptosis was rescued by the extrinsic overexpression of PLAGL2. Furthermore, PLAGL2 overexpression suppressed pro‐apoptotic Bax activation and Cyto C release from the mitochondria to the cytoplasm, but upregulated the expression of anti‐apoptotic Bcl‐2. These findings indicated that PLAGL2 was closely associated with apoptotic resistance. Several studies have revealed that PLAGL2 activates upstream and downstream signaling events by regulating the expression of ligands and receptors. For instance, Hu et al reported that the overexpression of PLAGL2 resulted in the upregulation of EGFR and its effector‐PI3K/AKT.^9^ Our results showed that PLAGL2 expression was positively correlated with the expression of C‐MET and downstream STAT3 and confirmed that the co‐activation of PLAGL2, p‐C‐MET, and p‐STAT3 existed in HCC. Taken together, PLAGL2 plays a critical role in tumor apoptosis resistance through C‐MET/STAT3 activation.

Despite the partial exploration of the tumorigenic role of PLAGL2, progress regarding the study of PLAGL2‐targeted drugs remains stagnant. Meanwhile, since inhibitors of PLAGL2 downstream effectors EGFR and C‐MET showed only modest effects in advanced‐stage HCC or phase 2 trials,^32^ the discovery of efficient PLAGL2 inhibitors is urgently needed for HCC targeted therapy. Computer‐aided drug design is time consuming and carries a high risk of failure; the repurposing of old drugs provides a simpler and more convenient way to address this issue.^33^ Several investigators have identified the potential correlation between selenium levels and the occurrence of HCC. Selenium, an essential component of a number of enzymes, is present in the amino acid selenocysteine (SeCys).^34^ Therefore, the regulation of selenium levels is predicted as a novel therapeutic option for HCC.^35^ There are several reports on the anticancer activity of Se‐containing compounds, including organic, inorganic, natural, and synthetic molecules.^36^ For instance, a selenium compound, PBISe, was found to be a chemotherapeutic agent in melanoma and human HCC.^37^ However, it is still necessary to explore and develop more selenium antitumor drugs with low toxicity and strong target efficacy. In the present study, we identified that SeS_2_, a compound typically found in lotion form and mainly used to treat seborrheic dermatitis and tinea versicolor, induced growth inhibition and mitochondrial apoptosis of HCC cells in vitro and in vivo in a PLAGL2‐dependent manner. The data obtained indicated that abundant PLAGL2 levels enhanced the growth inhibition and apoptosis induction effect of SeS_2_ on HCC cells. Furthermore, we evaluated the PLAGL2‐mediated effects of SeS_2_ in the SMMC‐7721 and Bel‐7402 xenograft models. SeS_2_ apparently deferred the growth of SMMC‐7721 and PLAGL/Bel‐7402 tumors by suppressing tumor volumes and masses, while maintaining the natural growth of mice. Similarly, PLAGL2 downregulation was observed in SeS_2_‐treated SMMC‐7721 xenograft samples.

We identified that C‐MET/STAT3 served as a novel downstream target of PLAGL2. The phosphorylation of C‐MET, a multifunctional tyrosine kinase receptor, can interact with several intracellular molecules, such as those involved in PI3K/AKT and Ras/MAPK signaling. In addition to the C‐MET/STAT3 cascade, both AKT/mTOR and MAPK pathways can mediate the aberrant growth and apoptosis resistance of cancer cells.^38^ Data presented here showed that SeS_2_ significantly inhibited the phosphorylation of C‐MET, STAT3, AKT, mTOR, MEK, ERK1/2, ERK5, JNK, and p38 in SK‐Hep‐1 and SMMC‐7721 cells. Notably, SeS_2_ treatment impeded the nuclear translocation of p‐STAT3 (Y705) and downregulated nuclear p‐STAT3 (Y705) expression.

The mitochondria‐located Bcl‐2 family of proteins plays crucial roles in mitochondrial‐mediated intrinsic apoptosis pathways, which maintain balance and prevent permeabilization of the outer membrane of the mitochondria.^39^ When the cell apoptosis process is initiated, the activation of pro‐apoptotic proteins, such as Bax, Bad, and Bak, leads to the release of Cyto C and AIF and activation of the Caspase cascade.^40^, ^41^ In this study, we observed a marked upregulation of Bad and Bak expression and the downregulation of Bcl‐2 and Mcl‐1 expression in SeS_2_‐treated SK‐Hep‐1 and SMMC‐7721 cells. In addition, SeS_2_ notably upregulated AIF expression and promoted Cyto C release from the mitochondria to the cytoplasm, which further activated the Caspase cascade and dose‐dependently increased PARP cleavage. These results indicated that SeS_2_ induced HCC cell apoptosis via intrinsic mitochondrial pathways.

**FIGURE 9:**
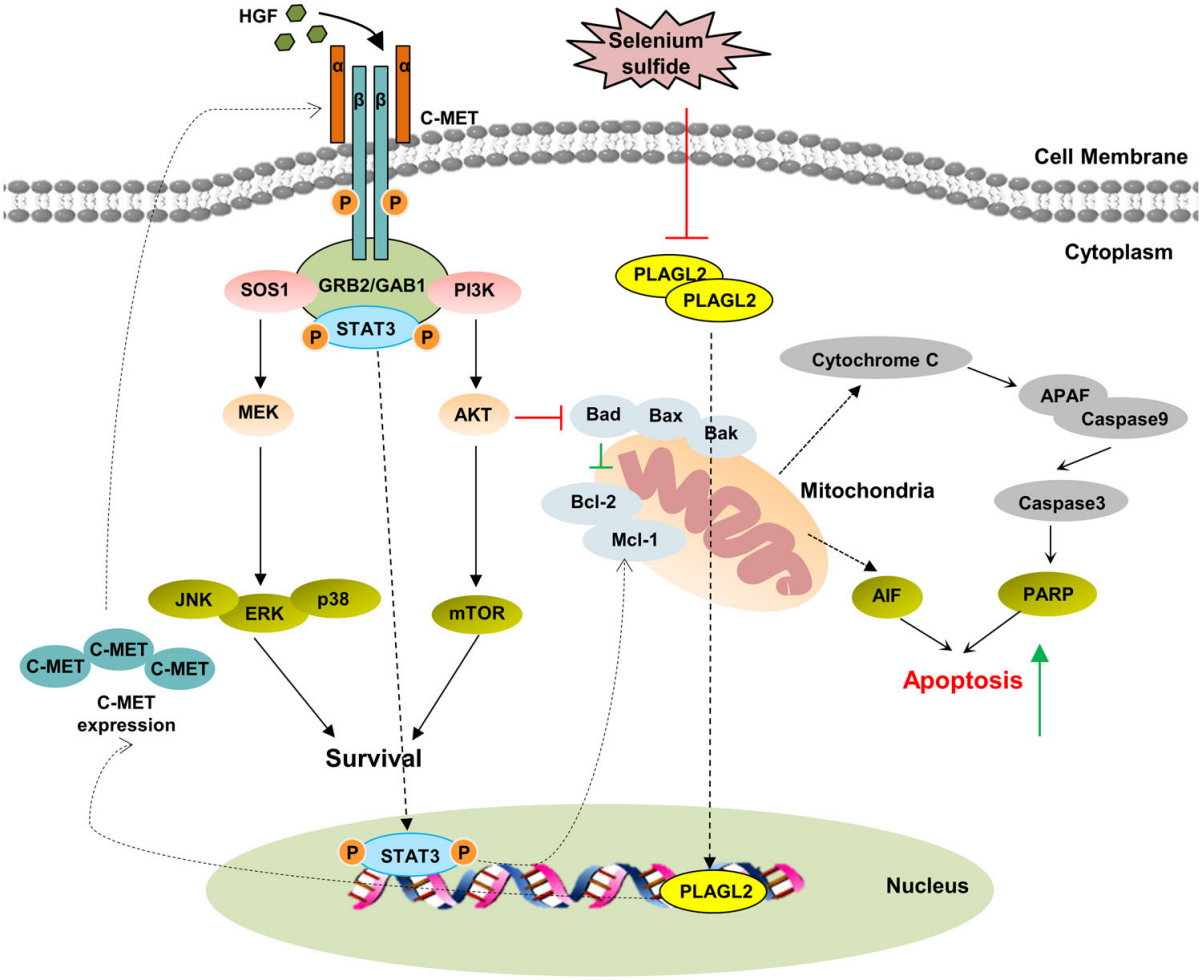
Schema of the potential mechanisms underlying the selenium sulfide‐induced proliferation inhibition and apoptosis of HCC cells. SeS_2_ inhibited the activity of PLAGL2 and downregulated the expression of the novel PLAGL2/C‐MET downstream in the STAT3, AKT/mTOR, and MAPK signaling pathways

In summary, we revealed the potential mechanism underlying PLAGL2‐mediated apoptosis resistance and the effect of SeS_2_ on HCC. As shown in Figure 9, the activation of PLAGL2 results in C‐MET overexpression and STAT3 activation, thus contributing to mitochondrial apoptosis resistance in HCC. SeS_2_ inhibited the growth of and induced apoptosis in HCC cells by inhibiting the expression of PLAGL2, thereby suppressing downstream C‐MET/STAT3, AKT/mTOR, and MAPK signaling. The BCL‐2/Cyto C/Caspase signaling pathway was stimulated by SeS_2_, which finally induced apoptosis in HCC cells. However, identification of the precise binding sites of SeS_2_ to PLAGL2 requires further study. The affinity and strength of interaction between proteins and small molecules can be investigated, and it can be determined whether the hydrophobic pocket is formed by residues on the PLAGL2 ligand.^41^ Further studies are needed to identify the specific mechanism and relationship between the inhibitory effect of SeS_2_ on C‐MET downstream pathways and its targeting effect on PLAGL2. In addition, since PLAGL2 functions as a transcription factor, whether SeS_2_ can affect the promoter activity of PLAGL2, and the genes it regulates need to be determined.^9^

## 5 CONCLUSIONS

Overall, our study identified PLAGL2 as a regulator of HCC apoptosis resistance via the C‐MET/STAT3 signaling axis. Given the critical role of PLAGL2 in HCC progression, we anticipate that our findings will provide more support for the development of drugs for HCC that specifically target PLAGL2. More importantly, we found that SeS_2_ could inhibit HCC cell proliferation and induce apoptosis as a potential PLAGL2 inhibitor. Moreover, further exploration and optimization of SeS_2_ would allow us to elucidate the mechanisms underlying the action of SeS_2_ and develop it as a new therapeutic strategy for malignant HCC.

## CONFLICT OF INTEREST

The authors declare that they have no competing interests.

## AUTHOR CONTRIBUTIONS

Tianfeng Yang designed the study, performed the experiments, and wrote the manuscript. Jian Huo and Rui Xu performed the experiments, analyzed, and interpreted the data. Qi Su and Wenjuan Tang interpreted the data and edited the manuscript. Dongdong Zhang, Man Zhu, and Yingzhuan Zhan helped with the study design and edited the manuscript. Bingling Dai and Yanmin Zhang conceived the project and designed the experiments, reviewed, and revised the manuscript. All authors read and approved the final version of the manuscript.

## Supporting information

Supporting Information

## Data Availability

All data generated or analyzed during this study are included in this published article (as well as in the accompanying Supporting Information).
