# Supplementary material for: Selenium sulfide disrupts the PLAGL2/C‐MET/STAT3‐induced resistance against mitochondrial apoptosis in hepatocellular carcinoma
Source: Clin Transl Med. 2021 Sep 15;11(9):e536. doi: 10.1002/ctm2.536 (PMC8441139; doi:10.1002/ctm2.536)
Supplement: Supplementary file 1 — Supporting Information [file CTM2-11-e536-s001.docx]

Selenium sulfide disrupts the PLAGL2/C-MET/STAT3-induced resistance against mitochondrial apoptosis in hepatocellular carcinoma

Tianfeng Yang^1,2^, Jian Huo^1,2^, Rui Xu^1,2^, Qi Su^1,2^, Wenjuan Tang^1,2^, Dongdong Zhang^1,2^, Man Zhu^1,2^, Yingzhuan Zhan^1,2^, Bingling Dai^1,2^**^*^**, Yanmin Zhang^1,2^**^*^**

1 School of Pharmacy, Health Science Center, Xi’an Jiaotong University, Xi’an 710061, P. R. China

2 State Key Laboratory of Shaanxi for Natural Medicines Research and Engineering Xi’an 710061 P. R. China

**Correspondence to:**

Dr. Bingling Dai and Yanmin Zhang

School of Pharmacy, Health Science Center, Xi’an Jiaotong University, Xi’an 710061, P. R. China

Tel.: +86 29 8265 6264; fax: +86 29 8265 5451

E-mail address: dbl1412@mail.xjtu.edu.cn (B.L. Dai)

zhang2008@mail.xjtu.edu.cn (Y.M. Zhang)

**1. Supporting Tables**

**2. Supporting Figures and Figure Legends**

**Table S1. The reagents have been used for this study.**

| **Name** | **Manufacturers** |
| --- | --- |
| Selenium sulfide (SeS2), Etoposide | Aladdin  Solarbio |
| CCCP, TMRE, Trypsin-EDTA, Penicillin-Streptomycin solution (100×), Puromycin |  |
| AZD1480 | TargetMol |
| AZD5363 | Meilunbio |
| AZD8330 | MedChemExpress |
| HGF | Sinobio |
| DMEM, RPMI-1640, MEM, PBS | HyClone |
| Opti-MEM | Gibco |
| FBS | ExCell Bio |
| MTT, DMSO, DAPI | Sigma-Aldrich |
| Crystal violet | Beijing Chemical Plant |
| Lipofectamine 2000 | Invitrogen |
| PE Annexin V Apoptosis Detection Kit I | BD Biosciences |
| Cy3-goat anti rabbit IgG, TUNEL detection Kit | Yeasen Biotechnology |
| Protease inhibitor and phosphatase inhibitor cocktails | Roche Technology |
| BCA protein assay reagent kit, RIPA lysis buffer, goat anti mouse IgG | Pioneer Biotechnology |
| Mitochondria /Nuclei Isolation Kit | Keygene Biotech |
| Enhanced Chemiluminescent (ECL) Plus Reagent kit | 4A Biotech |
| p-Stat3 (Tyr705), Stat3, p-AKT (S473), AKT, p-mTOR (S2448), p-MEK (T286), MEK, p-ERK (T202/Y204), ERK, p-ERK5 (Y220), p-JNK (Y185), JNK, p-p38 (T180), p38, Cleaved-PARP rabbit mAbs | Cell Signaling Technology |
| PLAGL2, Bax, Bcl-2, Cyto C, C-MET, mTOR, ERK5, Bak, Bad, Mcl-1, AIF, APAF-1, Cleaved-Caspase9, Caspase9, Cleaved-Caspase3, Caspase3, PARP1, VDAC, Histone H3 rabbit mAbs, GAPDH mouse mAbs, goat anti rabbit IgG | Protein Technology Group |
| p-C-MET (Y1349), GRB2, p-GAB1 (Y627), GAB1, SOS1 rabbit mAbs | ABclonal |
| FastPure Cell/Tissue Total RNA Isolation Kit, HiScript II One Step RT-PCR Kit, Taq Pro Universal SYBR qPCR Master Mix | Vazyme Biotech |
| Endo-free Plasmid Mini Kit I (50) | OMEGA bio-tek |
| PLAGL2-overexpressed bacterial strain | Genechem |
| shPLAGL2 bacterial strain | VectorBuilder |

**Table S2. Patient information of 15-paried HCC tissues.**

| **Patient number** | **Age** | **Sex** |
| --- | --- | --- |
| #1159850 | 58 | Male |
| #1172910 | 72 | Male |
| #1156004 | 63 | Female |
| #1138847 | 54 | Male |
| #1184610 | 80 | Male |
| #1157087 | 73 | Male |
| #1178840 | 58 | Male |
| #1172691 | 62 | Male |
| #1172262 | 56 | Male |
| #1172538 | 41 | Male |
| #1185953 | 56 | Female |
| #1183133 | 46 | Female |
| #1172393 | 58 | Male |
| #1109669 | 62 | Male |
| #1180415 | 63 | Female |

**Table S3. The primers have been used for RT-PCR.**

| **Gene** | | **Primer sequences** |
| --- | --- | --- |
| PLAGL2-F | 5’-CCAGAGCAGAGACCATATAG-3’ | |
| PLAGL2-R | 5’-AACATCTTATCACAGTACATACAC-3’ | |
| MET-F | 5'-GGACTTTTCCTGTGGCTGAAAAGG-3' | |
| MET-R | 5'-GGTAGTCTACAGATTCATTTG-3' | |
| STAT3-F | 5’-TGCCTTATCAGGGCTGGGATAC-3’ | |
| STAT3-R | 5’- GGGACCTTTAGACACGCAAGGA -3’ | |
| β-actin-F | 5’-GCGTGACATTAAGGAGAAG-3’ | |
| β-actin-R | 5’-GAAGGAAGGCTGGAAGAG-3’ | |

**Table S4. The IC_50_ value of** **selenium sulfide.**

| **Cell lines** | **IC_50_ (μM)** |
| --- | --- |
| SK-Hep-1 | 19.10 |
| SMMC-7721 | 23.81 |
| MHCC-97L | 36.95 |
| Huh-7 | 31.96 |
| Hep3B | 33.55 |
| Bel-7402 | 63.58 |
| Bel-7404 | 63.43 |
| HepG2 | 45.78 |
| L-02 | 68.43 |

**
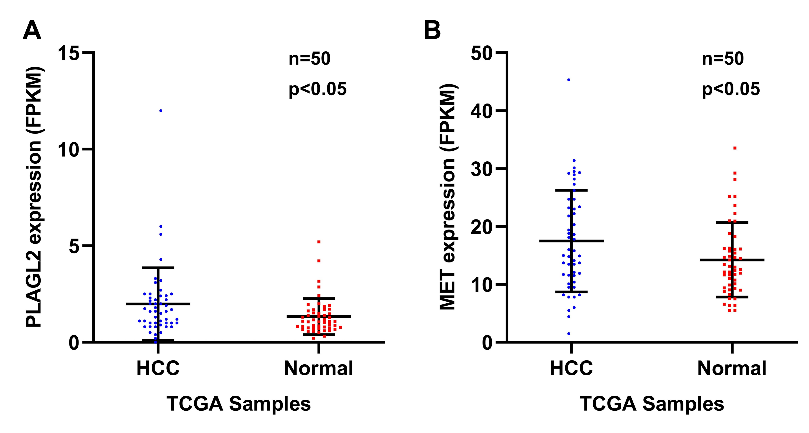
**

**FIGURE S1**

**FIGURE S1** PLAGL2 and MET are upregulated in human HCC tissues. (A) The expression of PLAGL2 was significantly higher in the 50 HCC tissues than in the 50-paired adjacent normal liver tissue samples in the TCGA database. (B) The expression of MET was significantly higher in the 50 HCC tissues than in the 50-paired adjacent normal liver tissue samples in the TCGA database.

**
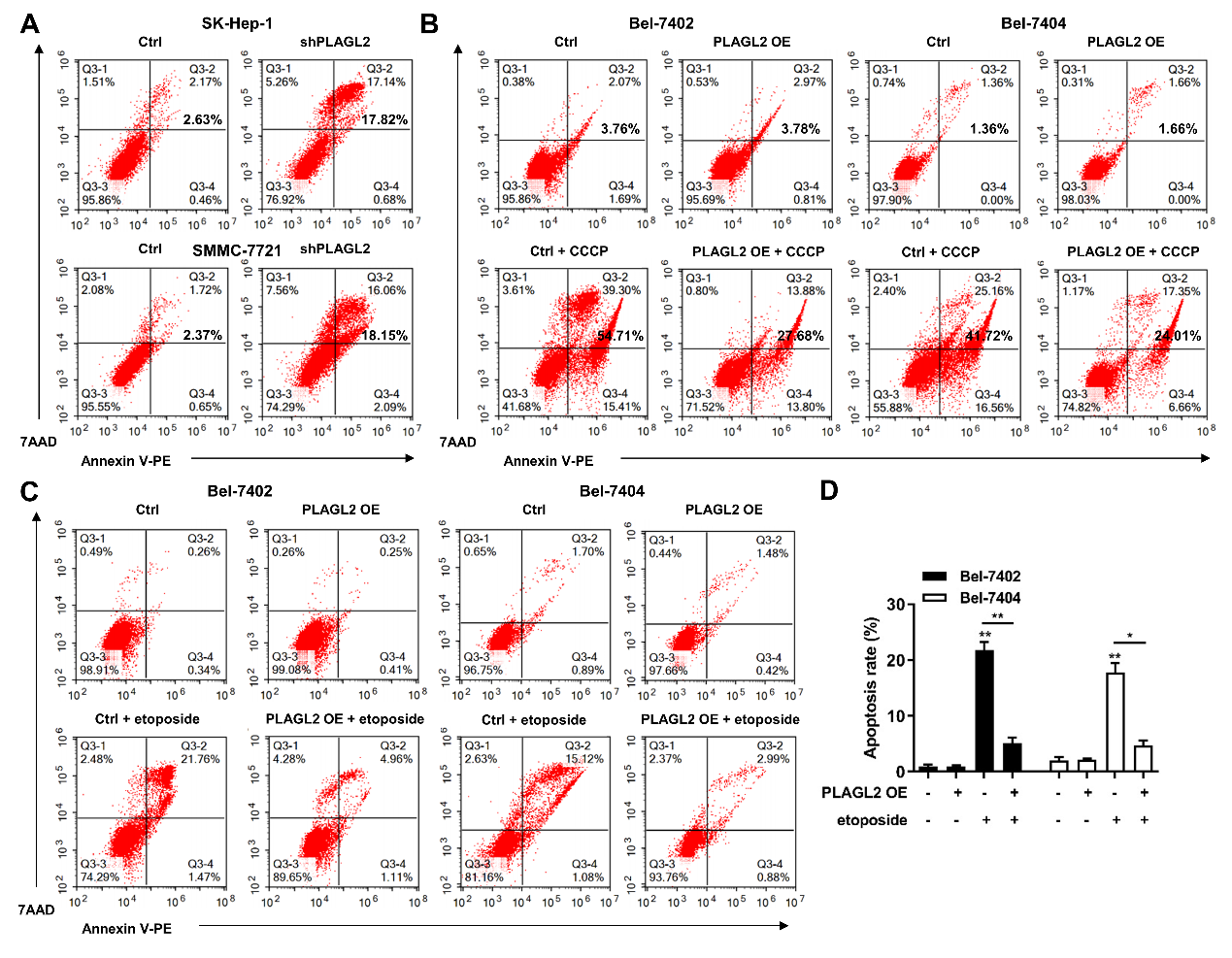
**

**FIGURE S2**

**FIGURE S2** PLAGL2 promotes resistance against CCCP-induced and etoposide-induced HCC cells apoptosis. (A) Representative results of cell apoptosis in wild-type control (Ctrl) and PLAGL2-knockdown (shPLAGL2) SK-Hep-1 and SMMC-7721 cells. (B) Representative results of cell apoptosis in CCCP-treated wild-type control (Ctrl) and PLAGL2-overexpressing (PLAGL2 OE) Bel-7402 and Bel-7404 cells. (C) Representative results of cell apoptosis in etoposide-treated Ctrl/Bel-7402, PLAGL2/Bel-7402, Ctrl/Bel-7404 and PLAGL2/Bel-7404 cells. (D) Quantification histogram of Figure S2C based on the percentage of apoptotic cells. Data are expressed as the means ± SEM (n = 3). **p* < 0.05, ***p* < 0.01 compared with the control group.

**
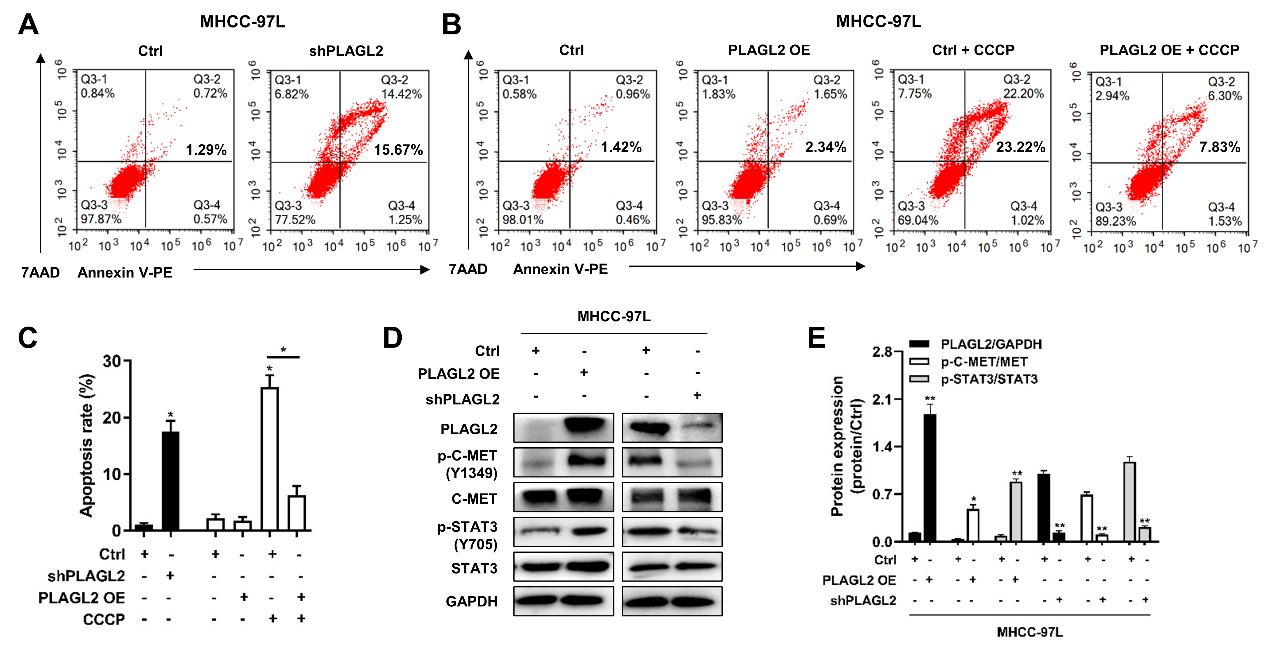
**

**FIGURE S3**

**FIGURE S3** PLAGL2 contributes to mitochondrial apoptosis resistance in MHCC-97L cells. (A) Representative results of cell apoptosis in WT/MHCC-97L and shPLAGL2/MHCC-97L cells. (B) Representative results of cell apoptosis in CCCP-treated Ctrl/MHCC-97L and PLAGL2/MHCC-97L cells. (C) Quantification histogram of Figure S3A and S3B based on the percentage of apoptotic cells. (D) C-MET and STAT3 expression following PLAGL2-knockdown and PLAGL2-overexpressing in MHCC-97L cells (Ctrl/MHCC-97L, shPLAGL2/ MHCC-97L, Ctrl/MHCC-97L, and PLAGL2/MHCC-97L) was detected at the total and phosphorylated protein levels by via western blotting. (E) Bar plot of Figure S3D. Data are expressed as the means ± SEM (n = 3). **p* < 0.05, ***p* < 0.01 compared with the control group.


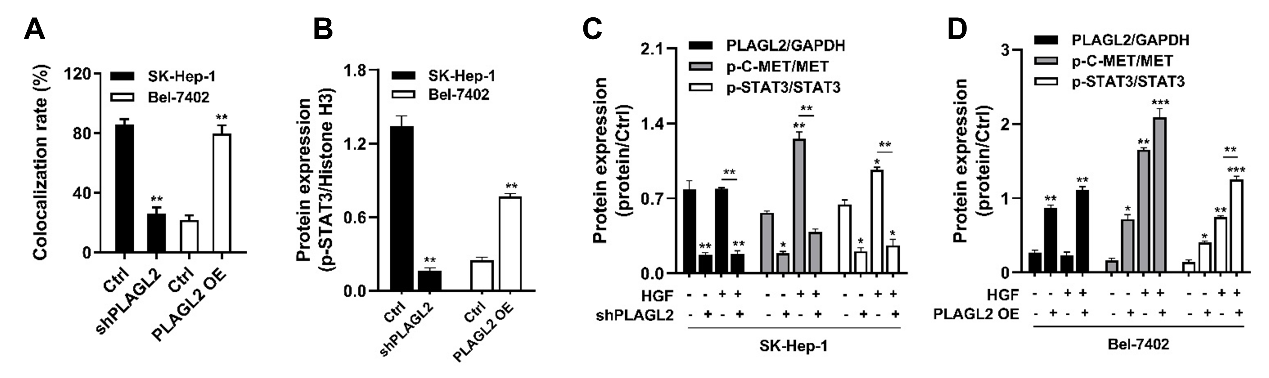


**FIGURE S4**

**FIGURE S4** PLAGL2 regulates the C-MET/STAT3 signaling axis. (A) Quantification histogram of Figure 3F and 3G based on the colocalization of blue and red fluorescence. (B) Bar plot of Figure 3H. (C) Bar plot of Figure 3I. (D) Bar plot of Figure 3J. Data are expressed as the means ± SEM (n = 3). **p* < 0.05, ***p* < 0.01, ****p* < 0.001 compared with the control group.


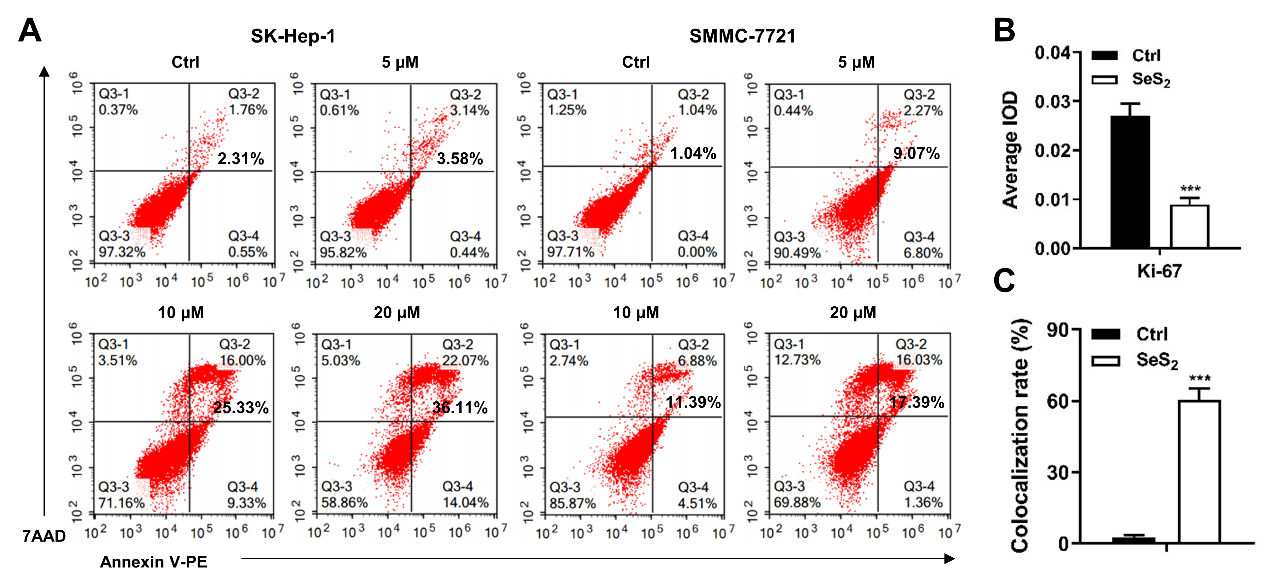


**FIGURE S5**

**FIGURE S5** Selenium sulfide induces apoptosis of HCC cells *in vitro* and *in vivo*. (A) Representative results of cell apoptosis in SK-Hep-1 and SMMC-7721 cells treated with SeS_2_ for 48 h. (B) Quantification of immunohistochemical analysis of Ki-67 expression (Figure 4M) in SMMC-7721 xenograft samples. (C) Quantification histogram of Figure 4N based on the colocalization of blue and green fluorescence. Data are expressed as the means ± SEM (n = 3). ****p* < 0.001 compared with the control group.


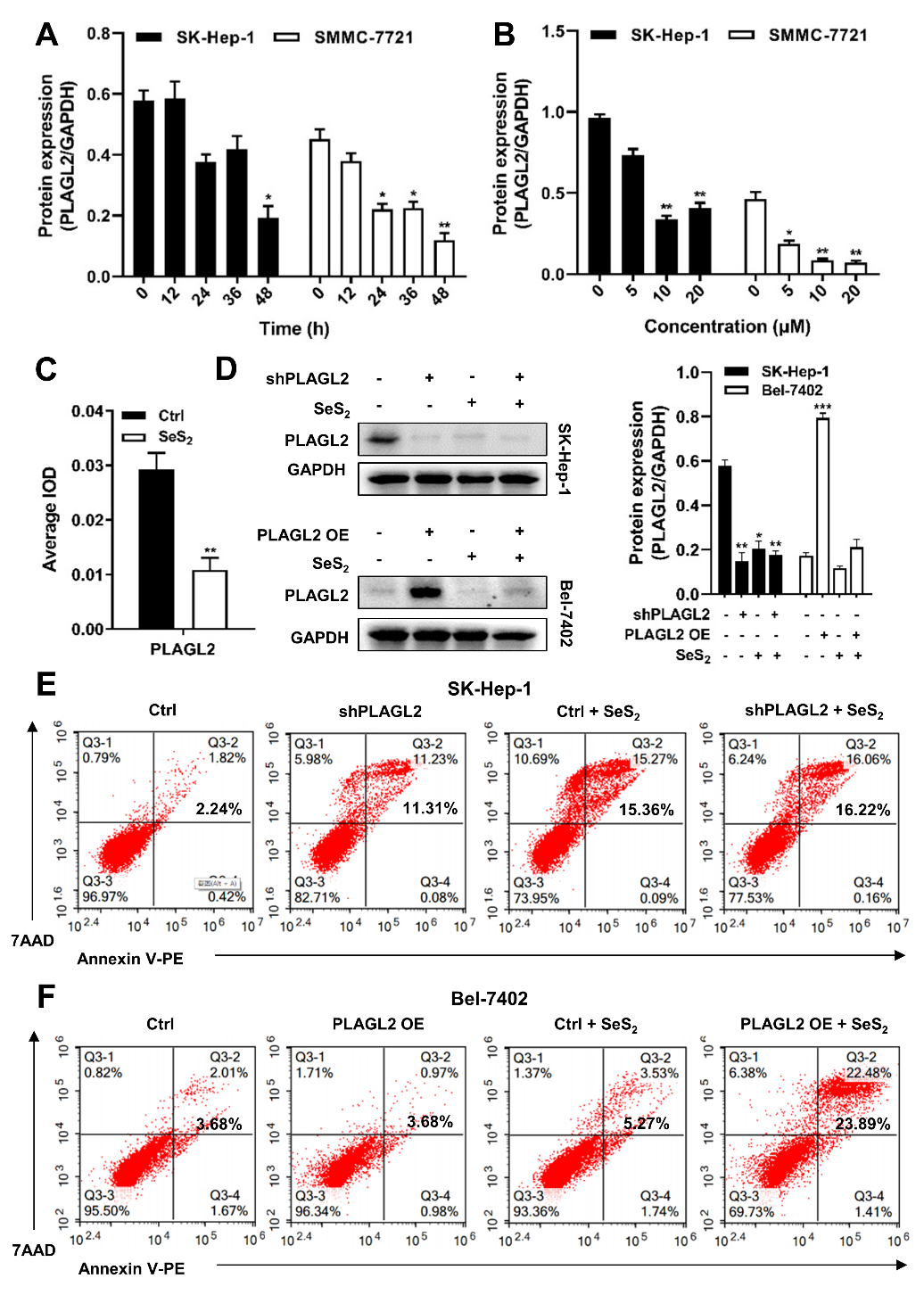


**FIGURE S6**

**FIGURE S6** PLAGL2 serves as a promising target for selenium sulfide. (A) Bar plot of Figure 5A. (B) Bar plot of Figure 5B. (C) Quantification histogram of Figure 5C. (D) Protein expression and quantification of PLAGL2 in SeS_2_-treated Ctrl/SK-Hep-1, shPLAGL2/SK-Hep-1, Ctrl/Bel-7402 and PLAGL2/Bel-7402 cells. (E, F) Representative results of cell apoptosis in SeS_2_-treated Ctrl/SK-Hep-1, shPLAGL2/SK-Hep-1, Ctrl/Bel-7402 and PLAGL2/Bel-7402 cells. Data are expressed as the means ± SEM (n = 3). **p* < 0.05, ***p* < 0.01, ****p* < 0.001 compared with the control group.

**
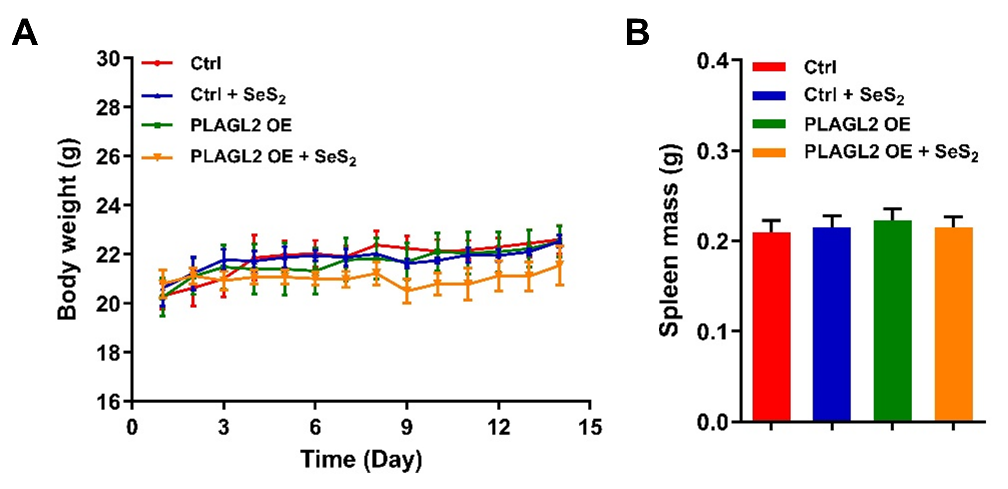
**

**FIGURE S7**

**FIGURE S7** Effects of selenium sulfide on mice body weight and spleen mass of Bel-7402 xenograft tumor models. (A) Body weight changes of mice harboring Ctrl/Bel-7402 and PLAGL2/Bel-7402 tumors. (B) Spleen mass of mice harboring Ctrl/Bel-7402 and PLAGL2/Bel-7402 tumors. Data are expressed as the means ± SEM (n = 4).


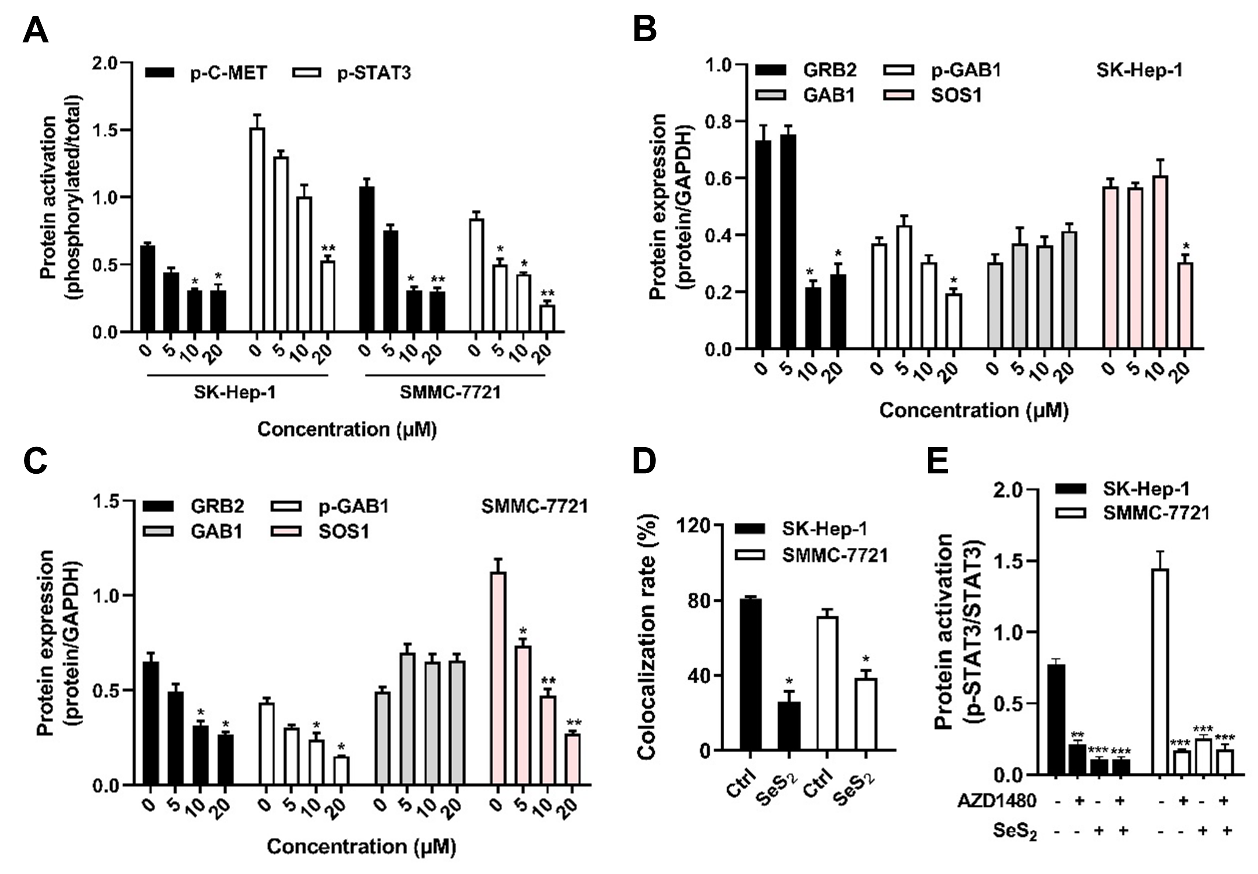


**FIGURE S8**

**FIGURE S8** Selenium sulfide inhibits the C-MET/STAT3 signaling axis. (A) Bar plot of Figure 6A and 6C. (B, C) Bar plot of Figure 6B. (D) Quantification histogram of Figure 6E and 6F based on the colocalization of blue and red fluorescence. (E) Bar plot of Figure 6H. Data are expressed as the means ± SEM (n = 3). **p* < 0.05, ***p* < 0.01, ****p* < 0.001 compared with the control group.


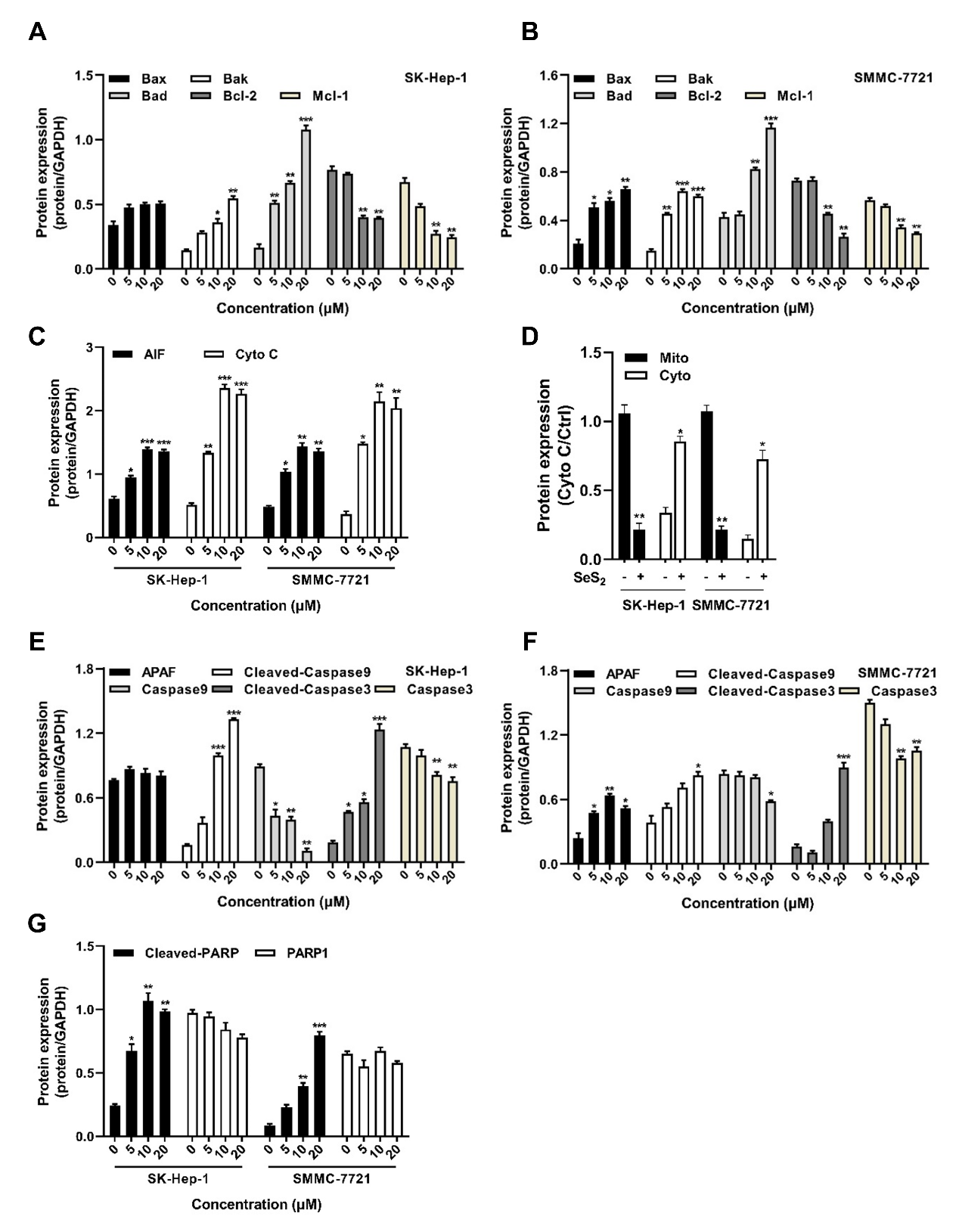


**FIGURE S9**

**FIGURE S9** Selenium sulfide triggers the intrinsic mitochondrial apoptosis pathway. (A, B) Bar plot of Figure 8A. (C) Bar plot of Figure 8B. (D) Bar plot of Figure 8C. (E, F) Bar plot of Figure 8D. (G) Bar plot of Figure 8E. Data are expressed as the means ± SEM (n = 3). **p* < 0.05, ***p* < 0.01, ****p* < 0.001 compared with the control group.
